# Tuning Receptor Properties of Metal–Amyloid Beta Complexes. Studies on the Interaction between Ni(II)–Aβ_5–9_ and Phosphates/Nucleotides

**DOI:** 10.1021/acs.inorgchem.1c03285

**Published:** 2021-12-08

**Authors:** Aleksandra Tobolska, Nina E. Wezynfeld, Urszula E. Wawrzyniak, Wojciech Bal, Wojciech Wróblewski

**Affiliations:** † Chair of Medical Biotechnology, Faculty of Chemistry, Warsaw University of Technology, Noakowskiego 3, Warsaw 00-664, Poland; ‡ Faculty of Chemistry, University of Warsaw, Pasteura 1, Warsaw 02-093, Poland; § Institute of Biochemistry and Biophysics, Polish Academy of Sciences, Pawińskiego 5a, Warsaw 02-106, Poland

## Abstract

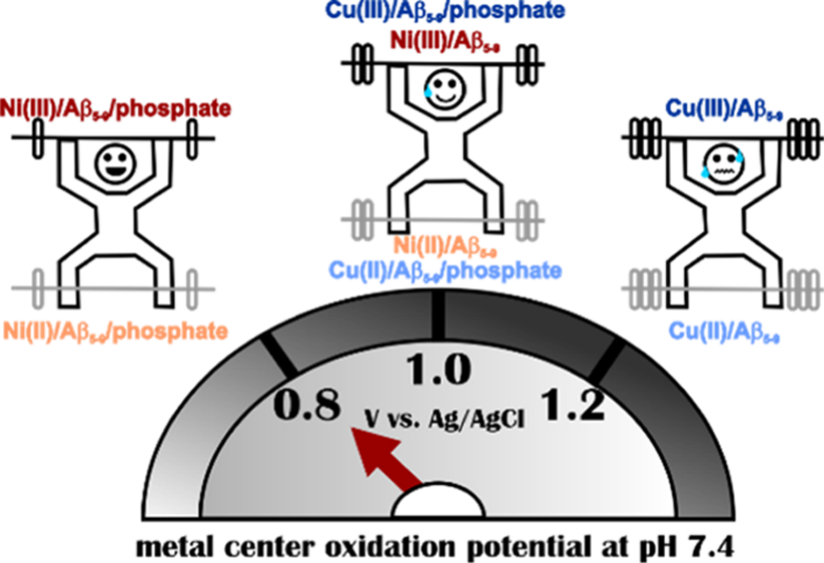

Amyloid-beta (Aβ) peptides, potentially relevant in the pathology of Alzheimer’s disease, possess distinctive coordination properties, enabling an effective binding of transition-metal ions, with a preference for Cu(II). In this work, we found that a N-truncated Aβ analogue bearing a His-2 motif, Aβ_5–9_, forms a stable Ni(II) high-spin octahedral complex at a physiological pH of 7.4 with labile coordination sites and facilitates ternary interactions with phosphates and nucleotides. As the pH increased above 9, a spin transition from a high-spin to a low-spin square-planar Ni(II) complex was observed. Employing electrochemical techniques, we showed that interactions between the binary Ni(II)–Aβ_5–9_ complex and phosphate species result in significant changes in the Ni(II) oxidation signal. Thus, the Ni(II)–Aβ_5–9_ complex could potentially serve as a receptor in electrochemical biosensors for phosphate species. The obtained results could also be important for nickel toxicology.

## Abstract 2

The switching of a metal center in the Aβ_5−9_ complex from Cu(II) to Ni(II) decreased its oxidation potential, retaining a good response toward phosphates and nucleotides. It raises the prospect of a metal−peptide receptor for phosphate species and could facilitate Ni(II) oxidation in complexes with His-2 peptides *in vivo*.

## Introduction

Peptides and proteins are common and efficient metal-binding ligands. Many of them, including amyloid-β (Aβ) related to Alzheimer’s disease, are rich in histidine (His) residues. The location of His residues in the peptide chain and the nature of the metal ion result in a variety of coordination properties and thermodynamic stabilities of the resulting chelates.^1^ The presence of the His residue near the free N-terminus of peptides and proteins prompts high affinities of these ligands to transition-metal ions such as Cu(II) and Ni(II) through unique His-2 (XH) and His-3 (XZH) metal binding motifs (X stands for any amino acid residue and Z for any residue excluding Pro).^2,3^ Peptides with these motifs have been employed in biological and medicinal applications, including copper sensing,^4^ biomolecule (*e.g.*, DNA) degradation,^5^ biological imaging,^6^ and ROS quenching.^7^ Most studies published in the literature have focused on the His-3 motif,^8^ but the His-2 one could be even more appealing due to the labile coordination sites and, in consequence, the propensity to form ternary complexes.^9−13^ This feature seems to be of great importance for selective recognition of biomolecules such as anions.

Recently, we demonstrated that a Cu(II) complex with an N-truncated Aβ analogue Aβ_5–9_ (Arg–His–Asp–Ser–Gly–NH_2,_ where −NH_2_ stands for C-terminal amidation) is a promising molecular receptor for phosphates at physiological conditions.^14^ In this complex, Cu(II) is bound to the N-terminal amine, the imidazole of His2, and the intervening deprotonated amide N^–^, forming a 3N complex with the fourth labile equatorial position that could be occupied by a water molecule, a buffer molecule, or another peptide group.^12^ We proved that phosphates could replace the water molecule in the 3N Cu(II)–Aβ_5–9_ complex, where the Arg1 side chain stabilizes the interaction between phosphates and the complex by intermolecular forces.

The activity of peptides and proteins with the His-2 motif has been related to their Cu(II) complexes, with GHK as the most notable case.^15,16^ In contrast, nickel is considered mostly a toxic metal for the human body, and therefore, Ni(II) complexes of the His-2 peptides have been less studied. Ni(II)–peptide complexes generally are characterized by a higher pK of the metal-driven amide deprotonation compared to their Cu(II) counterparts, and consequently, the complexation processes are shifted to higher pH. Additionally, Ni(II) complexes exhibit high-spin/low-spin equilibria. In complexes with the His-2 peptides, the high-spin, hexa-, or penta-coordinated species prevail mostly at pH 6–7, while the low-spin complexes structurally analogous to Cu(II) species are generally formed at higher pH values.^17,18^ These features can be expected to affect the properties of metal–peptide complexes as anion receptors. Moreover, the oxidation of the metal center for Ni(II) complexes is expected to occur at lower potentials than for the Cu(II)–peptide complexes^19,20^ due to a smaller gain in the crystal field stabilization energy for the transition from d^8^ Ni(II) to d^7^ Ni(III) compared to the transition from d^9^ Cu(II) to d^8^ Cu(III). Accordingly, it could improve the recognition of a given analyte by the metal–peptide receptor in electrochemical biosensors. Thus, we decided to explore the Ni(II)–Aβ_5–9_ complex to improve the recognition of phosphates by the His-2 complexes.

In this work, we investigated Ni(II) coordination to Aβ_5–9_ and the electrochemical properties of the resulting complexes. Next, we explored the ability of Ni(II)–Aβ_5–9_ to bind biologically relevant anions, including phosphates and nucleotides, with a focus on the redox properties of such ternary systems. On this basis, we determined the sensitivity and selectivity of the Ni(II)–Aβ_5–9_ complex toward selected analytes. Potentiometry and spectroscopy [UV–vis and circular dichroism (CD)] were employed to describe the structures and stabilities of Ni(II)–Aβ_5–9_ binary complexes, while their redox properties and the effects of anions were investigated by voltammetry [cyclic voltammetry (CV) and differential pulse voltammetry (DPV)].

## Experimental Methods

### Chemicals and Reagents

Fmoc amino acids were purchased from Novabiochem. The TentaGel S RAM resin was obtained from RAPP Polymere. Diethyl ether was purchased from Chempur. Acetonitrile and trifluoroacetic acid (TFA) were received from Avantor and Merck, respectively. Potassium nitrate, nitric acid, hydrochloric acid, potassium hydroxide, sodium hydroxide, nickel(II) chloride hexahydrate (99.999% trace-metal basis), sodium chloride, sodium acetate anhydrous, sodium phosphate monobasic, adenosine 5′-monophosphate (AMP) sodium salt, cytidine 5′-monophosphate (CMP) disodium salt, uridine 5′-monophosphate (UMP) disodium salt, adenosine 5′-triphosphate (ATP) disodium salt, cytidine 5′-triphosphate (CTP) disodium salt, and uridine 5′-triphosphate (UTP) trisodium salt dihydrate were received from Sigma-Aldrich. Nickel(II) nitrate hexahydrate (purity 99.999%) and dimethylformamide were purchased from Carl Roth. Sodium sulfate anhydrous was supplied by Fluka. All solutions were prepared daily with deionized water (the resistivity was 18 MΩ·cm) passed through an Arium mini Lab Water System (Sartorius). In order to prevent nucleotide hydrolysis, the pH of each stock solution was adjusted to 7.0–7.4 and the stock solutions were kept on ice during experiments. The glassware was rinsed with 6 M HNO_3_, followed by deionized water before use to avoid metal ion contamination.

### Peptide Synthesis

The Aβ_5–9_ peptide (Arg–His–Asp–Ser–Gly–NH_2_) was synthesized on a Prelude peptide synthesizer (Protein Technologies, Inc. Tucson, AZ) according to the Fmoc strategy.^21^ The peptide crude was purified by HPLC on a Breeze system (Waters) equipped with a Vydac semipreparative column (5 mm particle size, 10 × 250 mm). The mobile phase was a linear gradient of solution B [0.1% (v/v) TFA/90% (v/v) acetonitrile/9.9% (v/v) water] in solution A [0.1% (v/v) TFA/99.9% (v/v) water]. The purity of the lyophilized peptide was verified by a Q-Tof Premier mass spectrometer (Waters) exhibiting correct molecular masses.

### Potentiometric Titrations

Potentiometric titration of the Aβ_5–9_ peptide and its Ni(II) complexes was performed on a 907 Titrando automatic titrator (Metrohm, Herisau, Switzerland) using a Biotrode combined glass electrode (Metrohm, Herisau, Switzerland) calibrated daily by nitric acid titrations. 100 mM NaOH (carbon dioxide-free) was used as a titrant. 1.5 mL samples were prepared in a 96 mM KNO_3_/4 mM HNO_3_ solution. Complex formation was studied using 1:0.33, 1:0.45, and 1:0.9 peptide-to-Ni(II) molar ratios. All experiments were performed under argon at 25 °C in the pH range of 2.7–11.5. The obtained data were analyzed using the SUPERQUAD and HYPERQUAD programs.^22,23^

### UV–Vis and CD Titrations

The pH-metric titrations were recorded at 25 °C on a Varian Cary 50 spectrophotometer (Agilent) over the spectral range 200–900 nm and on a J-815 CD spectropolarimeter (JASCO) covering the spectral range of 228–850 nm with a 1 cm-path quartz cuvette (Helma). NiCl_2_ solution was added to the samples to reach a 1:0.9 peptide-to-metal ratio. Titrations were performed in water by adding small amounts of concentrated NaOH solution. The pH stability was checked for each titration point and adjusted with small amounts of concentrated NaOH or HCl solutions. Dilutions were included in the calculations.

### Voltammetric Experiments

Electrochemical measurements were performed using a CHI 1030 potentiostat (CH Instrument, Austin, USA) in a three-electrode arrangement: glassy carbon electrode (GCE, BASi, Ø = 3 mm) as the working electrode, an Ag/AgCl, 3 M KCl electrode (MINERAL, Poland) as the reference (electrolytic bridge filled with 100 mM KNO_3_), and a platinum wire as the counter electrode (MINERAL, Poland). The working electrode was sequentially polished with 1.0 μm and 0.3 μm alumina powder on a polishing cloth to the mirror-like surface, followed by 1 min ultrasonication in deionized water. All electrochemical measurements were carried out in 100 mM KNO_3_ at room temperature under an argon atmosphere. The concentration of peptide was 0.5 mM, whereas the Ni(NO_3_)_2_ solution was added to the samples to obtain a 1:0.9 peptide/Ni(II) molar ratio to avoid Ni(OH)_2_ precipitation.

The concentration of anions (in selectivity tests) and nucleotides was 10-fold higher than the initial concentration of the peptide. The pH was adjusted by adding small amounts of concentrated KOH or HNO_3_ solutions and controlled using a SevenCompact pH meter (Mettler-Toledo) with an InLab Micro Pro combination pH electrode (Mettler-Toledo). The applied techniques were CV and DPV. During CV measurements, various scan rates (*v*) were applied, whereas the following parameters were used in DPV: a pulse amplitude of 0.05 V, a pulse width of 0.1 s, a sample width of 0.005 s, and a pulse period of 1s.

## Results and Discussion

### Coordination of Aβ_5–9_ by Ni(II)

A set of potentiometric titrations of the Aβ_5–9_ peptide in the absence and in the presence of Ni(II) ions were performed to determine the stability of the Ni(II)–Aβ_5–9_ complexes. The values of cumulative protonation constants (given as logarithms of cumulative association constants log β) and the respective values of stepwise constants (given as negative logarithms of stepwise acidic dissociation constants p*K*_a_) for the Aβ_5–9_ peptide are collected in Table 1. These values are in agreement with a previous determination.^12^ As described in Table 1 in a descending order, they are associated with the protonation/deprotonation of the N-terminal amine, the imidazole ring of His2, and the carboxyl group of Asp3.

**Table 1:** Cumulative Protonation and Stability Constants (log β) and Stepwise Constant (p*K*_a_) Values for Aβ_5–9_ (L) and Its Ni(II) Complexes at *I* = 0.1 M (KNO_3_) and 25 °C^a^

species	log β^b^	p*K*_a_	assignment	coordination mode
[HL]^1+^	7.37(1)	7.37	Arg1 N-term.	
[H_2_L]^2+^	13.51(1)	6.13	His-2	
[H_3_L]^3+^	16.67(1)	3.17	Asp3	
[NiH_–1_L]^1+^	–0.90(1)	5.96^c^		3N [N^am^, N^–^, N^im^] +H_2_O
[NiH_–2_L]^0^	–9.74(1)	8.84		3N [N^am^, N^–^, N^im^] + OH^–^
[NiH_–3_L]^1–^	–19.49(1)	9.75		4N [N^am^, 3 N^–^]

a *K*_a_ stands for an acidic dissociation constant.

b For a general species Ni_*p*_H_*q*_L_*r*_, the stability constant β is defined as β_NipHqLr_ = [Ni_*p*_H_*q*_L_*r*_]/([Ni]^*p*^ × [H^+^]^*q*^ × [L]^*r*^). Standard deviations on the last digits provided by HYPERQUAD, given in parentheses, represent the statistical errors of constant determinations. Assignments are based on literature values.^12,18,24^

c Calculated based on the distribution diagram presented in Figure 1.

In the next step, we calculated the stability constants of Ni(II)–Aβ_5–9_ complexes. Their values for the most reliable coordination model are depicted in Table 1, whereas the respective species distribution is shown in Figure 1. We distinguished three major species. The formation of the first one, [NiH_–1_L]^1+^, started at a pH around 5 and reached a maximum at a physiological pH around 7.4. The second one, [NiH_–2_L]^0^, appeared at a pH above 7, followed by [NiH_–3_L]^1–^ that emerged at a pH above 8.5. The applied model of Ni(II)–Aβ_5–9_ coordination was further verified by CD and UV–vis titration results derived from spectra that are shown in Figure 2. The pH dependence of absorbance at characteristic wavelengths was merged with the species distribution, as shown in Figure 1. Such a high degree of convergence of spectroscopic and potentiometric data confirms the proposed coordination model. It also allowed us to estimate the spectroscopic parameters for individual complexes given in Table 2 (see also spectral patterns in Supporting Information, Figure S1).

**Figure 1:**
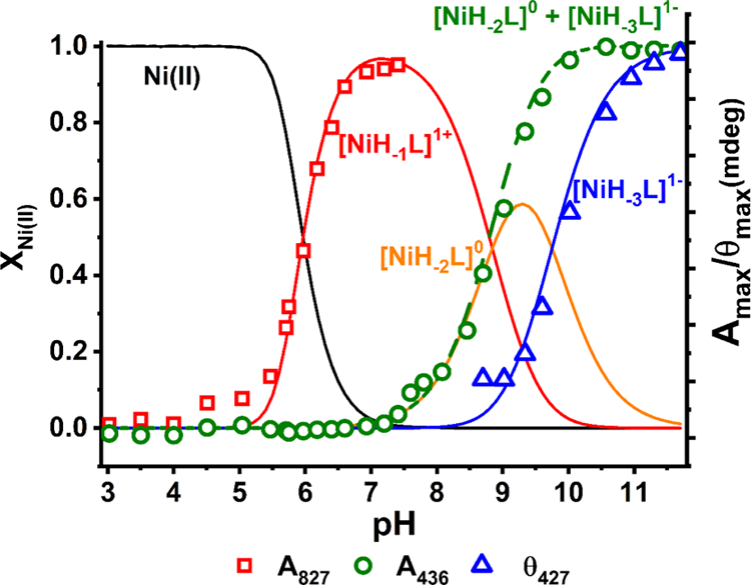
Species distribution calculated for 0.9 mM Ni(II) and 1.0 mM Aβ_5−9_ using stability constants presented in Table 1, with selected spectroscopic parameters overlaid.

**Figure 2:**
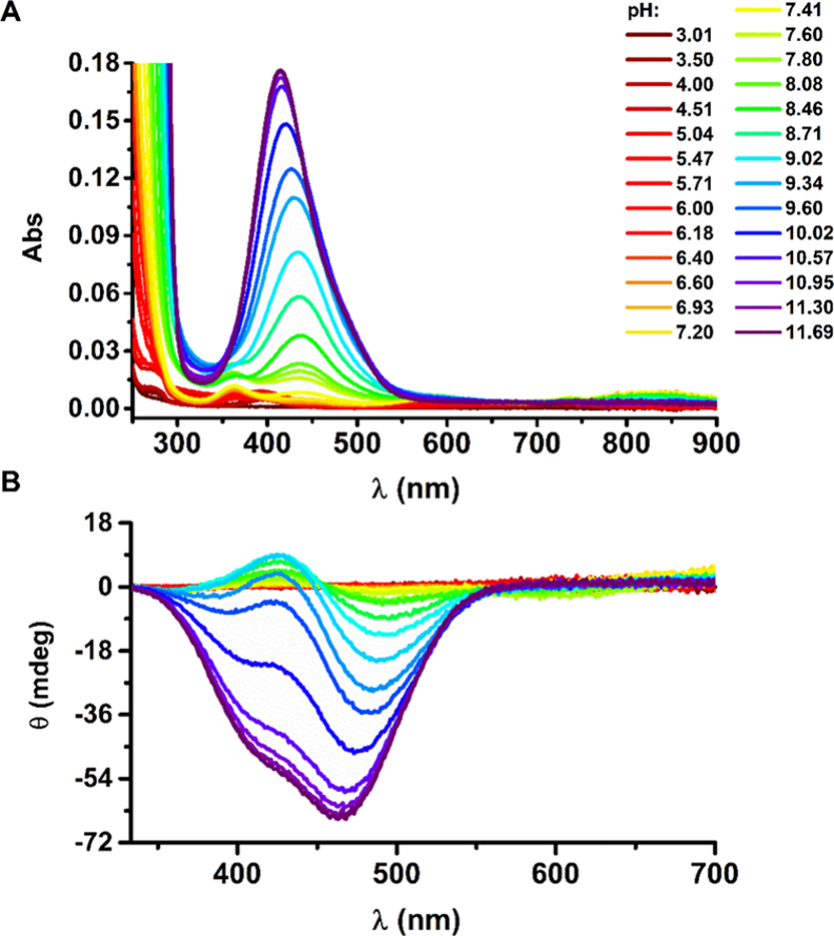
UV–vis (A) and CD (B) spectra of titrations of 1.0 mM Aβ_5–9_ and 0.9 mM NiCl_2_ with NaOH coded with rainbow colors from red to purple, pH range: 3.0–11.7.

**Table 2:** Spectroscopic Parameters of the Ni(II)–Aβ_5–9_ Complexes

	UV–vis	CD
species	λ_max_/nm (ε/ M^−1^ cm^−1^)	λ_ext_/nm (Δε/ M^−1^ cm^−1^)
[NiH_–1_L]^1+^	827 (9.6)	235 (−3.30)
	586 (6.0)	
	363 (12.2)	
[NiH_–2_L]^0^	438 (135.6)	488 (−1.34)
		426 (0.19)
[NiH_–3_L]^1–^	415 (195.8)	464 (−2.20)
		415 (−1.64) sh^a^

a sh stands for a spectral shoulder.

We observed three low-intensity bands at UV–vis spectra for [NiH_–1_L]^1+^ at 363, 586, and 827 nm (Table 2, Figure 2A), significantly blue-shifted from those registered for Ni(II) aqua ions (without peptide) (see Supporting Information, Figure S1). It demonstrates that [NiH_–1_L]^1+^ is a high-spin, octahedral complex, where Ni(II) is bound to several nitrogen ligands.^25^ Simultaneously, the [NiH_–1_L]^1+^ stoichiometry indicates that its formation was enabled by deprotonation of four peptide groups, including at least one that does not undergo deprotonation for the peptide alone at the studied pH range 2.7–11.7. Based on literature data,^18^ we propose that for [NiH_–1_L]^1+^, the Ni(II) ion is bound through the Arg1 N-terminal amine, His2 amide, and His2 imidazole nitrogen, forming a 3N complex (3N [N^am^, N^–^, N^im^] + H_2_O) with a deprotonated Asp3 carboxyl side chain. The remaining positions in the coordination sphere are likely occupied by a water molecule and also by the Asp3 carboxyl or the carbonyl oxygen.^26^ The CD spectra (Table 2) reveal only one negative CT band at 235 nm described in the literature as the N^am^ → Ni(II) CT band (with a possible contribution of the imidazole nitrogen),^27^ confirming the proposed coordination mode.

The subsequent deprotonation resulted in the formation of the [NiH_–2_L]^0^ species that caused major changes in UV–vis and CD spectral patterns (Figure 2). The position of d–d bands at 438 nm for UV–vis and at 426/488 nm for CD (the positive/negative band) suggests that [NiH_–2_L]^0^ is a low-spin, square-planar complex (see Table 2). This transition from a high-spin to a low-spin Ni(II) complex could be associated with the deprotonation of a water molecule, resulting in the (3N [N^am^, N^–^, N^im^] + OH^–^) complex. The metal ion-induced water deprotonation is a common feature in Ni(II) peptidic complexes.^28^ It is also sufficient to trigger the spin transition.^25^ The proposed coordination pattern is confirmed by the alternate pattern of d–d bands in the CD spectra, associated with the presence of the six-membered chelate ring enabled by His imidazole coordination next to the metal ion–peptide nitrogen bond in Cu(II) and Ni(II) complexes.^17,29,30^

The [NiH_–3_L]^1–^ species occurs under the most alkaline conditions and causes further changes in CD spectra with two negative signals, a band at 464 nm and a shoulder at 415 nm, of much higher intensity compared to that of [NiH_–2_L]^0^, as well as a 23 nm blue shift of the d–d band in the UV–vis spectra. The stoichiometry of this final complex associated with the change of sign of CD d–d bands to uniformly negative indicates the coordination of the Arg1 amine and three subsequent peptide nitrogens (His2, Asp3, and Ser4).^31,32^ It is a result of the pH-driven deprotonation of the second and the third amides and the removal of the imidazole of His2 from the Ni(II) coordination sphere with the formation of a 4N [N^am^, 3N^–^] complex.

The ligand exchange kinetics of Ni(II) complexes, especially that associated with the spin transition, is usually a slow process, which requires the rearrangement of the electronic structure and ligand exchange.^33^ Therefore, we checked the kinetics of the spin transition in the studied system. The UV–vis signals of Ni(II)/Aβ_5–9_ at pH 7.4 appeared just after reagent mixing/pH adjustment and were stable overnight (including the band of the low-spin complex at 438 nm), as shown in Supporting Information, Figure S2. Likewise, the pH change from 7.0 to 10.5 led to substantial changes in the UV–vis spectra immediately upon the pH adjustment, and only a slight increase of about 5% was noted during the overnight incubation (see Supporting Information, Figure S3). This confirms that the kinetics of the formation of low-spin Ni(II)–Aβ_5–9_ according to the applied procedure (with no external buffer but with the pH adjustment and pH control before each measurement) should not significantly alter our analysis.

### Electrochemistry of Ni(II)–Aβ_5–9_ Binary Complexes

**Figure 3:**
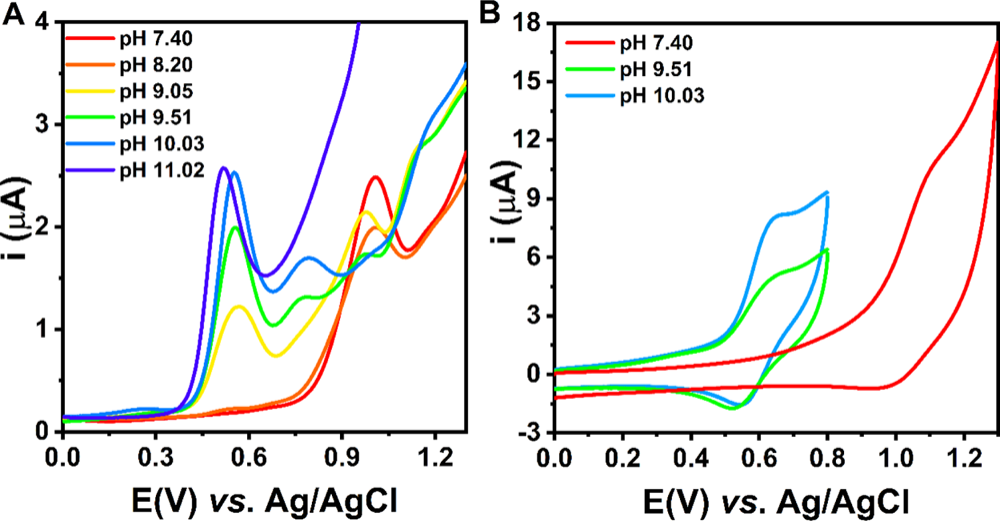
DPV (A) and selected CV (B) curves of the titration of Ni(II)–Aβ_5–9_ (0.9:1.0 ratio) with KOH, recorded in 100 mM KNO_3_, scan rate: *v* = 0.1 V/s. The CV curves at pH 9–10 in panel B were recorded in a shorter potential range to avoid the effect of the other processes at high potentials on the Ni(III) reduction in the reverse scan.

The cyclic voltammograms of Ni(II)–Aβ_5–9_ recorded at pH 7.4 are depicted in Figures S4 and S5 (Supporting Information) and in Figure 3 (red curves). As expected, no electrochemical reduction of the complex metal center was observed (Figure S4). The CV curves recorded in the positive potential range showed the oxidation of Ni(II) at pH 7.4 with a very weak signal of Ni(III) reduction on the reverse scan (Figure 3B). An increase in the scan rate slightly intensified the Ni(III) reduction (see Supporting Information, Figure S5). However, the separation of anodic and cathodic peaks (Δ*E*_p_ = 90 mV) indicates a slow electron transfer. In the literature, reversible or quasi-reversible Ni(II)/Ni(III) redox processes (Δ*E*_p_ = 83 mV) were observed for ATCUN (His-3) complexes, or generally, for square-planar complex structures.^19^ They are associated with a strong stabilization of electrogenerated Ni(III) species in these chelates provided mostly by deprotonated peptide nitrogens. Ni(II)–Aβ_5–9_ forms predominantly an octahedral complex at pH 7.4 with just a few percent of the [NiH_–2_L]^0^ square-planar species present in equilibrium. The high-spin complex requires a significant structural rearrangement on the pathway to Ni(III) as similar Ni(III)–peptide complexes are described as tetragonal with the approximately square-planar arrangement of coordinated peptide nitrogens.^34,35^ However, there are also known examples of Ni(III) square-pyramidal complexes formed by NiSOD-inspired peptides.^36^ The Ni(III)–peptide species demonstrate pro-oxidative properties, often causing peptide oxidation^37−39^ and likely resulting in the almost irreversible Ni(II) oxidation for Ni(II)–Aβ_5–9_ at slower potential sweeps. Considering a slow electron transport in the Ni(II)/Ni(III) cycle and associated difficulties in the analysis of the CV data, the DPV technique was mainly used in further studies.

To fully characterize the redox properties of the binary complex, we analyzed the pH dependence of the Ni(II)–Aβ_5–9_ electrochemical signal (Figure 3A). The Ni(II) oxidation potentials for the individual species are provided in Table 3. As pH increased above 7.4, the intensity of the signal related to the Ni(II)/Ni(III) couple (around 1.0 V *vs* Ag/AgCl) decreased. At a pH of about 9.5, we observed three independent peaks likely associated with the metal center oxidation in the three coordinating forms that coexisted in the mixture ([NiH_–1_L]^1+^, [NiH_–2_L]^0^, and [NiH_–3_L]^1–^). At high alkaline pH values, only one peak appeared (around 0.5 V *vs* Ag/AgCl), in agreement with the predominant role of a square-planar (4N [N^am^, 3 N^–^]) system of (5-5-5)-membered chelate rings in Ni(II) coordination. A similar Ni(II) oxidation potential value (around 0.6 V *vs* Ag/AgCl) was reported for Ni(II) complexes of tetrapeptides consisting of glycine and alanine residues,^20^ confirming the proposed coordination mode for the [NiH_–3_L]^1–^ species of Ni(II)–Aβ_5–9_. As demonstrated previously, such systems are most suitable to stabilize Ni(III) species.^20,40^ On the other hand, Bossu and Margerum showed that the contribution of histidine residues in the Ni(II) coordination and a reduced number of amide donors shifted the Ni(II) oxidation potential toward more positive values. For example, a shift to around 0.7 V versus Ag/AgCl was noticed for the ATCUN complexes due to the presence of a (5-5-6)-membered chelate ring and/or an occurrence of the π-back-bonding in addition to the σ-bonding between imidazole nitrogen and Ni(II) ions, while the shift to around 0.6–0.7 V versus Ag/AgCl was reported for tripeptides of noncoordinating side chains as a result of a lower number of amide groups that stabilize Ni(III) due to their stronger electron donor properties compared to amine or carboxylate groups.^20^ Both these effects are also expected to increase the potential of Ni(II) oxidation for the [NiH_–2_L]^0^ species of Ni(II)–Aβ_5–9_ compared to [NiH_–3_L]^1–^. Moreover, CV data indicate a quasi-reversible Ni(II) oxidation (Δ*E*_p_ = 77 mV; Figure 3B, a light-blue line), confirming the Ni(III) stabilization in the 4N chelate.

**Figure 4:**
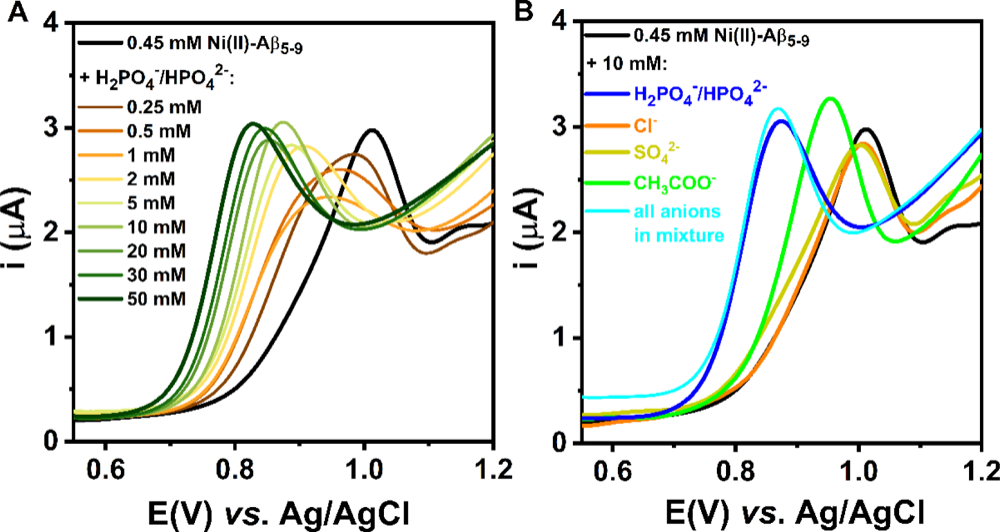
DPV curves of the titration of 0.45 mM Ni(II)–Aβ_5–9_ (0.9:1.0 ratio) with NaH_2_PO_4_/Na_2_HPO_4_ (A) and DPV curves obtained for 0.45 mM Ni(II)–Aβ_5–9_ alone and after the addition of 10 mM of selected anions (B), recorded in 100 mM KNO_3_ at pH 7.4.

**Table 3:** Oxidation Potential Values Determined from the DPV Curves of the Ni(II)–Aβ_5–9_ Complexes

Ni(II)–Aβ_5–9_	E_Ni(II)/Ni(III)_ (V) *vs* Ag/AgCl ± SD
[NiH_–1_L]^1+^	1.016 ± 0.008
[NiH_–2_L]^0^	0.790 ± 0.005
[NiH_–3_L]^1–^	0.512 ± 0.012

The observed shift of the Ni(II) oxidation toward less positive potentials for higher pH values is also consistent with an expected impact of the complex charge on the redox process. More negative charge of the complex should favor the electron release and, consequently, result in the lower potential of Ni(II) oxidation^41,42^ as observed for the studied Ni(II)–Aβ_5–9_ system (see Table 3).

### Interactions with Phosphate Anions and Nucleotides

Based on the potentiometric (Figure 1) and the voltammetric (Figure 3) results, the pH value of 7.4 was chosen for further studies due to its close resemblance to the physiological pH and the predominance of the octahedral form of the Ni(II)–Aβ_5–9_ complex that may possess more than one available analyte binding site. The investigation of the interaction between Ni(II)–Aβ_5–9_ and phosphate species by spectroscopic methods was very problematic due to the low intensity of the basal signal of the metal complex and small spectral changes in the course of the titration with phosphates, as shown in Supporting Information, Figure S6. These results suggest a very weak interaction (with the estimated *K*_a_ about 16 ± 5 M^–1^), even weaker than those reported between phosphates and Cu(II)–Aβ_5–9_ (*K*_a_ about 50 M^–1^).^14^ However, in the previous paper, we showed that electrochemical methods could be very useful in the studies of similar systems^14^ as the redox activity could be triggered even by small changes in the coordination sphere. Therefore, we mostly employed CV and DPV techniques in this work to check if phosphates could also alter Ni(II)/Ni(III) oxidation.

We started with a titration of the binary Ni(II)–Aβ_5–9_ complex with phosphate solution, monitoring changes in DPV signals (Figure 4A). As the H_2_PO_4_^–^/HPO_4_^2–^ concentration increased, the oxidation peak of the Ni(II)/Ni(III) couple successively shifted toward less positive values. At the end of the titration, for about a 100-fold excess of phosphate over Ni(II)–Aβ_5–9_, the oxidation signal occurred at a potential lower by ca. 175 mV than that of the source Ni(II)–Aβ_5–9_ complex. The large facilitation of the Ni(II)/Ni(III) redox process in the presence of phosphate species illustrates that the phosphate buffer used routinely in many experiments in the concentration range of 20–100 mM could drastically impact the results and analysis (*e.g.*, during the investigation of an extracellular process in a 50 mM phosphate buffer, whereas the extracellular phosphate concentration is about 1 mM^43^). On the other hand, the most extensive changes were observed up to 5 mM of phosphates (see also Supporting Information, Figure S7), which is consistent with the limit value of the reported intracellular range of phosphate concentration of 0.5–5 mM.^44^ As such, the pattern of the response of Ni(II)–Aβ_5–9_ to phosphates is very promising for the phosphate sensing in the cell by the Ni(II)–Aβ_5–9_ complex.

Continuing the studies on the Ni(II)–Aβ_5–9_ complex as the potential molecular receptor, we also analyzed the response of the studied binary system toward selected anionic species. Therefore, we performed DPV measurements for the Ni(II)–Aβ_5–9_ solution containing interfering physiological and environmental anions (Figure 4B). The noteworthy changes for the Ni(II) oxidation signal were observed only in the presence of acetate and phosphate anions (Figure 4B, a green line and a blue line, respectively); for about a 20-fold excess of the anions over Ni(II)–Aβ_5–9_, the Ni(II) oxidation potential decreased by 70 mV after the addition of acetate and even more, by 160 mV, for phosphate (see Supporting Information, Table S1). Nevertheless, the electrochemical response registered in a mixture of all tested anions (phosphate, chloride, sulfate, and acetate) indicated a significant selectivity toward the phosphate (Figure 4B, a cyan line).

The selectivity of receptors designed for phosphate anion recognition remains problematic, especially for inorganic phosphates over phosphorylated biomolecules. Thus, we tested the effect of the addition of various nucleotides on Ni(II)–Aβ_5–9_ redox properties. As nucleotides are electroactive species,^45^ we examined molecules whose oxidation potential is higher than 1.0 V (*vs* Ag/AgCl) at pH 7.4. We rejected the guanosine phosphates based on these criteria and chose adenosine, cytidine, and uridine phosphates for further studies.

It is well known from the literature that nucleotides coordinate metal ions. For complexes of all types of nucleotides, the metal ion is bound via the most basic terminal phosphate group and also in the purine nucleotides partially by nitrogen (N7) of the purine moiety.^46^ The control DPV curves for the respective Ni(II)–nucleotide binary systems did not yield peaks in the potential range from 0.5 to 1.0 V, and the only signals visible at the potential higher than 1.2 V were related to the nucleobase oxidation (see the DPV curves obtained for ATP in the absence and presence of Ni(II) ions in Supporting Information, Figure S8). Hence, this proved that the voltammetric signal observed around 0.8–1.0 V for the ternary system with nucleotides was not associated with the redox process of the binary Ni(II)–nucleotide complex.

**Figure 5:**
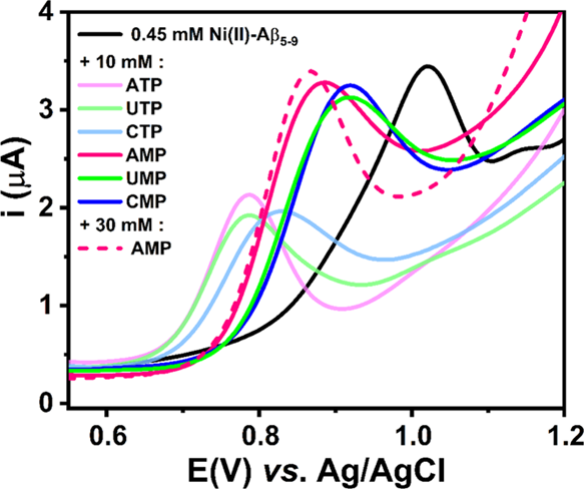
DPV curves obtained for 0.45 mM Ni(II)–Aβ_5–9_ alone (a black curve), after the addition of 10 mM nucleoside monophosphates (pink, blue, and green curves) or nucleoside triphosphates (faded pink, blue, and green curves), and after the addition of 30 mM adenosine monophosphate (a dashed pink curve). The measurements were done in 100 mM KNO_3_ at pH 7.4.

The presence of nucleotides in the Ni(II)–Aβ_5–9_ solution shifted the oxidation peak toward less positive potentials (Figure 5). It could be associated with the destabilization of the Ni(II) complex structure and the stabilization of the electrogenerated Ni(III) form in a ternary system facilitated by electrostatic and stacking interactions between the His imidazole ring and the nucleobase.^47^ However, in contrast to the previous study,^14^ the signal in the DPV curves occurred at different potentials for mono- and triphosphates (but only a slight shift of oxidation peak toward less positive potentials was observed with an increase of the AMP concentration from 10 mM to 30 mM). This can be explained by the ability of octahedral Ni(II) to interact with more than one nucleotide phosphate group due to the possible chelating effect and preference for phosphates over nucleobase nitrogens,^48^ in contrast to the more rigid four-coordinate planar structure of Cu(II)–Aβ_5–9_ complexes.

In accordance with the above considerations, the most facilitated (Δ*E*_ox_ ∼240 mV) Ni(II) oxidation process was noticed for ternary systems with nucleoside triphosphates (see Supporting Information, Table S1). This feature was evident for the obtained CV curves that showed a more reversible Ni(II)/Ni(III) redox process in the presence of nucleoside triphosphates (see Supporting Information, Figure S9). In addition, a signal intensity decrease observed after the triphosphate addition may be the result of slower diffusion of the complex to the electrode surface due to the increased size of the chelate.

### Comparison with the Ni(II) Complexes of Other His-2 Peptides

We compared the properties of the Ni(II)–Aβ_5–9_ complex to the literature data of Ni(II) complexes of His-2 peptides. However, it was not straightforward due to multiple binding models, different stoichiometries, and the formation of multimeric species proposed for these systems. Therefore, we calculated a pNi parameter according to the following equation
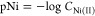
where C_Ni(II)_ is the concentration of Ni(II) aqua ions.

**Figure 6:**
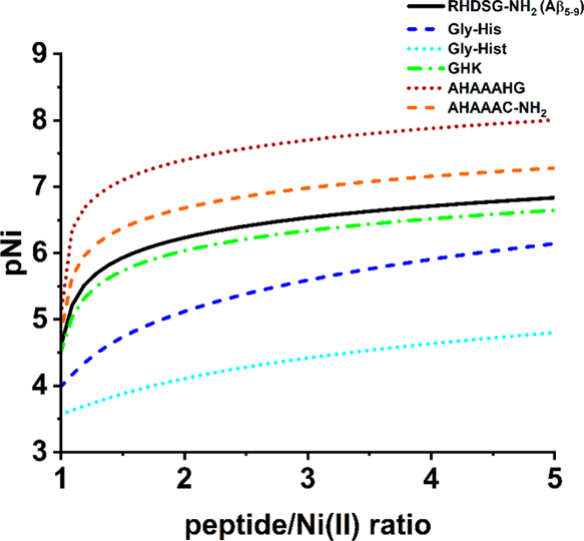
Dependence of pNi values on the peptide/Ni(II) molar ratio for different peptides calculated for 1 mM Ni(II) and 1–5 mM peptide at pH 7.4 based on potentiometric results obtained in this work for Aβ_5–9_ and published previously.^18,24,28,50,52^ Hist stands for histamine.

The simulation was performed by the Hyss software^49^ for 1 mM Ni(II), 1–5 mM peptide, and pH 7.4 (see Figure 6). The pNi values for the Ni(II)/Aβ_5–9_ system are in the middle of pNi values estimated for the previously studied Ni(II)/peptide system. Aβ_5–9_ has been found to have a higher affinity to Ni(II) ions compared to dipeptides (Gly–His and Gly–Hist),^28,50^ very similar to that of the tripeptide GHK,^18^ and lower than longer peptides with additional Ni(II) binding sites provided by further His or Cys residues.^24,51^ For all of them, the primary Ni(II) binding sites are believed to occur at the N-terminus composed of the N-terminal amine, the His2 amide, and the His2 imidazole forming the 3N complex. However, other than 1:1 stoichiometries have been proposed especially for shorter peptides due to the formation of oligomeric Ni(II):peptide species.^18,28,43^ The model proposed by us for Ni(II)/Aβ_5–9_ does not include multimeric species as their implementation did not improve the fitting. In accordance, the UV–vis titration of 0.9 mM Ni(II)/1 mM Aβ_5–9_ with the Aβ_5–9_ peptide did not show clear signs of such species (see Supporting Information, Figure S10). The addition of the peptide caused an increase in the intensity of the Ni(II) complex bands, but it was correlated with the general rise in neighboring signals of the high-intensity bands in the UV region due to the higher peptide concentration. Overall, we could not exclude the presence of multimeric species for Ni(II)/Aβ_5–9_, but their stability (if they exist) would be very low. Moreover, the presence of positively charged Arg1 should successfully hinder the interaction with other peptide molecules.

Finally, the highest pNi values were observed for the AHAAAHG/AHAAAC-NH_2_ peptides.^24,51^ The presence of another His/Cys residue at the C-terminus of AHAAAHG/AHAAC-NH_2_ peptides indeed increased the affinity to Ni(II) ions compared to Aβ_5–9_ or GHK. However, this amplification resulted likely from the interaction typical for His-2 peptides 3N chelating with the His/Cys residue further. Such interactions are not favored for our system as 3N [N^am^, N^–^, N^im^] + H_2_O is supposed to interact with phosphate and not compete with other groups of the peptide.

As such, the sequence of Aβ_5–9_ provides beneficial features compared to other peptides bearing the His-2 motif for its application as a Ni(II) complex in the receptor system of the sensor for the phosphate species. The Ni(II) affinity of Aβ_5–9_ should ensure a robust anchoring site for Ni(II) ions. On the other hand, the 3N [N^am^, N^–^, N^im^] + H_2_O species of the Ni(II)/Aβ_5–9_ complex, the core of the potential recognition of phosphates, is less susceptible to interaction with other peptide-derived groups of the intermolecular (as for Gly–His, Gly–Hist and also GHK at a slightly higher pH) or intramolecular character (as for peptides containing His/Cys residues at further positions).

### Effect of Altering the Metal Center of the Aβ_5–9_ Complex

At pH 7.4, the conditional stability constant of the Ni(II)–Aβ_5–9_ complex for 1 mM concentrations of reagents (*K*^c^ = 1.7 × 10^6^ M^–1^) is more than 5 orders of magnitude lower than for Cu(II)–Aβ_5–9_ (*K*^c^ = 5.8 × 10^12^ M^–1^). As such, the peptide Aβ_5–9_ would primarily form complexes with Cu(II) ions for the equimolar amounts of Cu(II)/Ni(II)/Aβ_5–9_ at pH 7.4 and generally at pH higher than 3.6, as calculated based on the potentiometric constants (see Supporting Information, Figure S11). Also, we checked the kinetics of the ligand exchange between Cu(II) and Ni(II) complexes adding an equimolar amount of Cu(II) ions to the Ni(II)–Aβ_5–9_ complex at pH 7.4. As shown in Supporting Information, Figure S12, the transfer was accomplished within the mixing time and the time of pH adjustment, confirming the dominance of Cu(II) complexes in the analyzed mixture.

On the other hand, the binding of Ni(II) to Aβ_5–9_ was strong enough to prevent the Ni(OH)_2_ precipitation noticed for other biomolecules, such as isomers of urocanic acid or peptide models of the prion protein.^53,54^ Thus, the stability of the Ni(II)–Aβ_5–9_ complex should be sufficient for its employment as a receptor for molecules of low Ni(II) affinity (such as phosphates).

Considering the electrochemical behavior of the studied system, the change of the metal center in the Aβ_5–9_ complex from Cu(II) to Ni(II) facilitated the oxidation of the metal center (Table 4) due to different geometries and stabilities of the complexes. This effect favors the application of the Ni(II)–Aβ_5–9_ complex in the receptor layer of an electrochemical biosensor. However, changes in the electrochemical response of such a complex upon phosphate anions addition were similar to those obtained for Cu(II)–Aβ_5–9_ (see Supporting Information, Figure S7 and Table 4). Thus, the sensitivity of anion recognition of both chelates is comparable. These results are related to similar Lewis acidities of Cu(II) and Ni(II) and suggest that despite potentially accessible axial sites in the Ni(II) complex, the phosphate anion’s favorable binding site is the equatorial position in the coordination sphere of the metal.

**Table 4:** Comparison of the Oxidation Potential Values *E*_M(II)/M(III)_ at pH 7.4 Determined from DPV Curves of Binary Ni(II)/Cu(II)–Aβ_5–9_ Complexes Alone and in the Presence of 10 mM Phosphates

	*E*_M(II)/M(III)_ (V) *vs* Ag/AgCl ± SD		
studied complex	binary complex	binary complex + 10 mM phosphates	Δ*E*_ox_ (mV)^a^
Ni(II)–Aβ_5–9_	1.016 ± 0.008	0.860 ± 0.002	–160
Cu(II)–Aβ_5–9_	1.204 ± 0.005	1.056 ± 0.006	–150

a Δ*E*_ox_ is the difference in the potential values in mV of Cu(II) or Ni(II) oxidation of the ternary system with phosphates and the binary complex round to the nearest tens.

**Figure 7:**
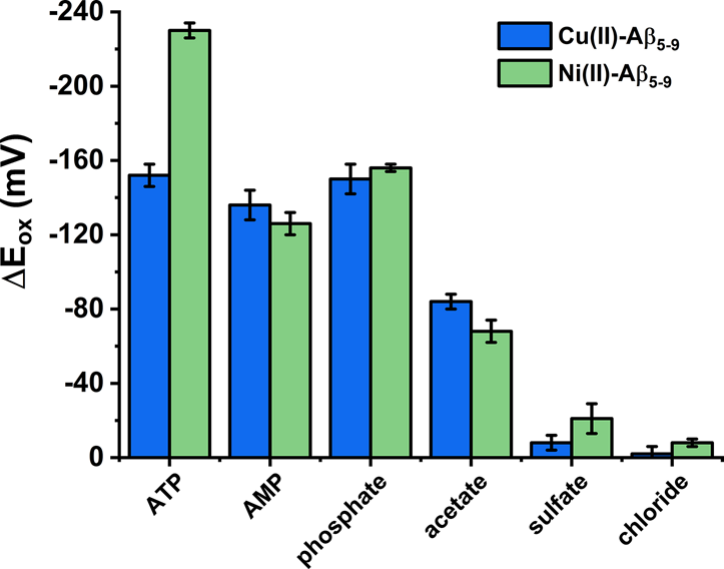
Comparison of selectivity of phosphate anion recognition at pH 7.4 between Ni(II)–Aβ_5–9_ and a Cu(II)–Aβ_5–9_ complex described previously.^14^ Δ*E*_ox_ is the difference in the potential values of Cu(II) or Ni(II) oxidation of the respective ternary system and the binary complex. Concentrations of reagents: 0.5 mM Aβ_5–9_, 0.45 mM Cu(II) or Ni(II), and 10 mM anions or nucleotides.

Nevertheless, an enhanced affinity for chloride and sulfate anions was observed for the Ni(II)–Aβ_5–9_ complex, which may be caused by the increased impact of electrostatic effects on the octahedral geometry of the binary complex (Figure 7).

The most significant difference in receptor properties of Cu(II)–/Ni(II)–Aβ_5–9_ is reflected in the interactions between nucleotides and the studied complexes. Cu(II)–Aβ_5–9_ is a square-planar Jahn–Teller distorted complex. Thus, it has a strong tendency to coordinate ligands in the equatorial position and prefer an interaction with the nucleotide’s most basic phosphate group, which resulted in a similar oxidation peak potential of Cu(II)–Aβ_5–9_ after AMP and ATP addition (Figure 7).^14^ The accompanying intermolecular forces, such as stacking interactions, will be preferred in the high-spin Ni(II)–Aβ_5–9_. The presence of the oxidation peak at a similar potential upon the addition of the same amount of phosphate anions and monophosphate nucleosides but at different potentials upon the addition of the same amount of triphosphates suggests a strong dependence of the Ni(II) oxidation potential on the number of phosphate groups in the phosphate species. Moreover, the signal related to Ni(II)/Ni(III) in that ternary system with Aβ_5–9_ occurs at the same potential value for pyrimidine and purine nucleotides, indicating irrelevance of the nucleobase type in such interactions.

### Biological Relevance

The facilitated Ni(II) oxidation of Ni(II) complexes of His-2 peptides in the presence of phosphates could also be very important for nickel toxicity. As phosphates are an integral part of the DNA structure, the DNA phosphate groups could attract the Ni(II) complexes of the His-2 peptides, increase the oxidative stress level provided by the Ni(II)/Ni(III) couple, and, in consequence, assist in the DNA cleavage. Previously, oxidative DNA cleavage was described mainly in the presence of Ni(II) complexes of His-3 peptides.^55−57^ The Ni(II) oxidation potential of such complexes was reported at about 0.7–0.9 V versus Ag/AgCl^20,34,58^ and was much lower than that of Ni(II) high-spin complexes with the His-2 peptides (about 1.0 V versus Ag/AgCl at pH 7.4). As shown by us, the interaction with phosphate could decrease the oxidation potential of the Ni(II) complexes of the His-2 peptides even to 0.8 V versus Ag/AgCl at pH 7.4. This value was reported for the known DNA cleavage catalysts − Ni(II) complexes of the His-3 peptides. The oxidative activity of Ni(II) complexes of the His-2 peptides has to be further proven by appropriate methods. However, the results obtained by us could be vital for studies of nickel toxicology.

## Conclusions

The Ni(II)–Aβ_5–9_ complex offers beneficial features as a potential receptor of phosphate anions. The sequence of the peptide with a histidine residue at the second position and with no further Ni(II)-binding sites should ensure a relatively strong Ni(II) binding (anchored at the His2 residue) with labile coordination sites eager to interact with phosphates. Such an interaction prompts a strong electrochemical response that could be valuable for phosphate sensing, including the detection of phosphate groups in more complex structures such as nucleotides.

## Supporting Information Available

Spectra of particular Ni(II) species; kinetic measurements of Ni(II)–Aβ_5–9_ formation; CV curves recorded in the Ni(II)/Aβ_5–9_ mixture toward negative values and toward more positive values at different scan rates; UV–vis titration of Ni(II)–Aβ_5–9_ with phosphates; comparison of the oxidation potentials for Ni(II) and Cu(II) complexes of Aβ_5–9_; DPV curves for ATP and Ni(II)/ATP mixtures; CV curves of Ni(II)–Aβ_5–9_ in the presence of nucleoside monophosphates and nucleoside triphosphates; UV–vis titration of Ni(II)–Aβ_5–9_ with Aβ_5–9_; theoretical ligand distribution in the mixture of Aβ_5–9_/Ni(II)/Cu(II); competition experiment between Ni(II)–Aβ_5–9_ and Cu(II) ions; and oxidation potential values for Ni(II)–Aβ_5–9_ in the presence of selected anions and nucleotides (PDF)

## References

[ref1] Sóvágó I.; Várnagy K.; Lihi N.; Grenács Á. Coordinating Properties of Peptides Containing Histidyl Residues. Coord. Chem. Rev. 2016, 327–328, 43–54. 10.1016/j.ccr.2016.04.015.

[ref2] Gonzalez P.; Bossak K.; Stefaniak E.; Hureau C.; Raibaut L.; Bal W.; Faller P. N-Terminal Cu-Binding Motifs (Xxx-Zzz-His, Xxx-His) and Their Derivatives: Chemistry, Biology and Medicinal Applications. Chem.—Eur. J. 2018, 24, 8029–8041. 10.1002/chem.201705398. 29336493 PMC6152890

[ref3] Bouraguba M.; Glattard E.; Naudé M.; Pelletier R.; Aisenbrey C.; Bechinger B.; Raibaut L.; Lebrun V.; Faller P. Copper-Binding Motifs Xxx-His or Xxx-Zzz-His (ATCUN) Linked to an Antimicrobial Peptide: Cu-Binding, Antimicrobial Activity and ROS Production. J. Inorg. Biochem. 2020, 213, 111255 10.1016/j.jinorgbio.2020.111255. 32980641

[ref4] Torrado A.; Walkup G. K.; Imperiali B. Exploiting Polypeptide Motifs for the Design of Selective Cu(II) Ion Chemosensors. J. Am. Chem. Soc. 1998, 120, 609–610. 10.1021/ja973357k.

[ref5] Agbale C. M.; Cardoso M. H.; Galyuon I. K.; Franco O. L. Designing Metallodrugs with Nuclease and Protease Activity. Metallomics 2016, 8, 1159–1169. 10.1039/c6mt00133e. 27714031

[ref6] Miyamoto T.; Fukino Y.; Kamino S.; Ueda M.; Enomoto S. Enhanced Stability of Cu2+-ATCUN Complexes under Physiologically Relevant Conditions by Insertion of Structurally Bulky and Hydrophobic Amino Acid Residues into the ATCUN Motif. Dalton Trans. 2016, 45, 9436–9445. 10.1039/c6dt01387b. 27184978

[ref7] Hureau C.; Sasaki I.; Gras E.; Faller P. Two Functions, One Molecule: A Metal-Binding and a Targeting Moiety to Combat Alzheimer’s Disease. ChemBioChem 2010, 11, 950–953. 10.1002/cbic.201000102. 20401891

[ref8] Harford C.; Sarkar B. Amino Terminal Cu(II)- and Ni(II)-Binding (ATCUN) Motif of Proteins and Peptides: Metal Binding, DNA Cleavage, and Other Properties. Acc. Chem. Res. 1997, 30, 123–130. 10.1021/ar9501535.

[ref9] Kotuniak R.; Fra̧czyk T.; Skrobecki P.; Płonka D.; Bal W. Gly-His-Thr-Asp-Amide, an Insulin-Activating Peptide from the Human Pancreas Is a Strong Cu(II) but a Weak Zn(II) Chelator. Inorg. Chem. 2018, 57, 15507–15516. 10.1021/acs.inorgchem.8b02841. 30480433

[ref10] Bossak K.; Mital M.; Poznański J.; Bonna A.; Drew S.; Bal W. Interactions of α-Factor-1, a Yeast Pheromone, and Its Analogue with Copper(II) Ions and Low-Molecular-Weight Ligands Yield Very Stable Complexes. Inorg. Chem. 2016, 55, 7829–7831. 10.1021/acs.inorgchem.6b01441. 27476515

[ref11] Bossak-Ahmad K.; Wiśniewska M. D.; Bal W.; Drew S. C.; Frączyk T. Ternary Cu(II) Complex with GHK Peptide and Cis-Urocanic Acid as a Potential Physiologically Functional Copper Chelate. Int. J. Mol. Sci. 2020, 21, 6190 10.3390/ijms21176190. PMC7503498 32867146

[ref12] Wezynfeld N. E.; Tobolska A.; Mital M.; Wawrzyniak U. E.; Wiloch M. Z.; Płonka D.; Bossak-Ahmad K.; Wróblewski W.; Bal W. Aβ5-x Peptides: N-Terminal Truncation Yields Tunable Cu(II) Complexes. Inorg. Chem. 2020, 59, 14000–14011. 10.1021/acs.inorgchem.0c01773. 32924459 PMC7539298

[ref13] Hureau C.; Eury H.; Guillot R.; Bijani C.; Sayen S.; Solari P.-L.; Guillon E.; Faller P.; Dorlet P. X-ray and Solution Structures of Cu^II^GHK and Cu^II^DAHK Complexes: Influence on Their Redox Properties. Chem.—Eur. J. 2011, 17, 10151–10160. 10.1002/chem.201100751. 21780203

[ref14] Tobolska A.; Wezynfeld N. E.; Wawrzyniak U. E.; Bal W.; Wróblewski W. Copper(II) complex of N-truncated amyloid-β peptide bearing a His-2 motif as a potential receptor for phosphate anions. Dalton Trans. 2021, 50, 2726–2730. 10.1039/d0dt03898a. 33576751

[ref15] Pickart L.; Margolina A. Regenerative and Protective Actions of the GHK-Cu Peptide in the Light of the New Gene Data. Int. J. Mol. Sci. 2018, 19, 1987 10.3390/IJMS19071987. PMC6073405 29986520

[ref16] Pickart L.; Thaler M. M. Tripeptide in Human Serum Which Prolongs Survival of Normal Liver Cells and Stimulates Growth in Neoplastic Liver. Nat. New Biol. 1973, 243, 85–87. 4349963

[ref17] Kozłowski H.; Bal W.; Dyba M.; Kowalik-Jankowska T. Specific Structure-Stability Relations in Metallopeptides. Coord. Chem. Rev. 1999, 184, 319–346. 10.1016/S0010-8545(98)00261-6.

[ref18] Conato C.; Kozłowski H.; Swiatek-Kozłowska J.; Młynarz P.; Remelli M.; Silvestri S. Formation Equilibria of Nickel Complexes with Glycyl-Histidyl-Lysine and Two Synthetic Analogues. J. Inorg. Biochem. 2004, 98, 153–160. 10.1016/j.jinorgbio.2003.09.010. 14659644

[ref19] Neupane K. P.; Aldous A. R.; Kritzer J. A. Metal-Binding and Redox Properties of Substituted Linear and Cyclic ATCUN Motifs. J. Inorg. Biochem. 2014, 139, 65–76. 10.1016/j.jinorgbio.2014.06.004. 24980953 PMC4119837

[ref20] Bossu F. P.; Margerum D. W. Electrode potentials of nickel(III)- and nickel(II)-peptide complexes. Inorg. Chem. 1977, 16, 1210–1214. 10.1021/ic50171a047.

[ref21] Chan W. C.; White P. D. Fmoc Solid Phase Peptide Synthesis: A Practical Approach; Chan W. C., White P. D., Eds.; Oxford University Press: New York, 2000; pp 41–76.

[ref22] Gans P.; Sabatini A.; Vacca A. SUPERQUAD: An Improved General Program for Computation of Formation Constants from Potentiometric Data. J. Chem. Soc., Dalton Trans. 1985, 6, 1195–1200. 10.1039/DT9850001195.

[ref23] Gans P.; Sabatini A.; Vacca A. Investigation of Equilibria in Solution. Determination of Equilibrium Constants with the HYPERQUAD Suite of Programs. Talanta 1996, 43, 1739–1753. 10.1016/0039-9140(96)01958-3. 18966661

[ref24] Grenács Á.; Kaluha A.; Kállay C.; Jószai V.; Sanna D.; Sóvágó I. Binary and Ternary Mixed Metal Complexes of Terminally Free Peptides Containing Two Different Histidyl Binding Sites. J. Inorg. Biochem. 2013, 128, 17–25. 10.1016/j.jinorgbio.2013.07.008. 23911567

[ref25] Kurzak B.; Bal W.; Kozłowski H. Nickel(II) Complexes of Hydroxamic Analogues of Aminoacids. J. Inorg. Biochem. 1990, 38, 9–16. 10.1016/0162-0134(90)85002-E.

[ref26] Kozłowski H.; Lebkiri A.; Onindo C. O.; Pettit L. D.; Galey J.-F. The Influence of Aspartic or Glutamic Acid Residues in Tetrapeptides on the Formation of Complexes with Nickel(II) and Zinc(II). Polyhedron 1995, 14, 211–218. 10.1016/0277-5387(94)00239-B.

[ref27] Bal W.; Jeżowska-Bojczuk M.; Kasprzak K. S. Binding of Nickel(II) and Copper(II) to the N-Terminal Sequence of Human Protamine HP2. Chem. Res. Toxicol. 1997, 10, 906–914. 10.1021/tx970028x. 9282840

[ref28] Gajda T.; Henry B.; Delpuech J.-J. Potentiometric and Spectroscopic Study of Nickel(II) and Cobalt(II) Complexes of Histamine-Containing Dipeptides. Inorg. Chem. 1995, 34, 2455–2460. 10.1021/ic00113a029.

[ref29] Kurowska E.; Bonna A.; Goch G.; Bal W. Salivary Histatin-5, a Physiologically Relevant Ligand for Ni(II) Ions. J. Inorg. Biochem. 2011, 105, 1220–1225. 10.1016/J.JINORGBIO.2011.06.002. 21741339

[ref30] Zawisza I.; Mital M.; Polkowska-Nowakowska A.; Bonna A.; Bal W. The Impact of Synthetic Analogs of Histidine on Copper(II) and Nickel(II) Coordination Properties to an Albumin-like Peptide. Possible Leads towards New Metallodrugs. J. Inorg. Biochem. 2014, 139, 1–8. 10.1016/J.JINORGBIO.2014.05.011. 24950384

[ref31] Pettit L. D.; Pyburn S.; Kozłowski H.; Decock-Le Reverend B.; Liman F. Co-Ordination of Nickel(II) Ions by Angiotensin II and Its Peptide Fragments. A Potentiometric, Proton Nuclear Magnetic Resonance and Circular Dichroism Spectroscopic Study. J. Chem. Soc., Dalton Trans. 1989, 8, 1471–1475. 10.1039/DT9890001471.

[ref32] Sigel H.; Martin R. B. Coordinating Properties of the Amide Bond. Stability and Structure of Metal Ion Complexes of Peptides and Related Ligands. Chem. Rev. 1982, 82, 385–426. 10.1021/cr00050a003.

[ref33] Bannister C. E.; Raycheba J. M. T.; Margerum D. W. Kinetics of Nickel(II) Glycylglycyl-L-Histidine Reactions with Acids and Triethylenetetramine. Inorg. Chem. 2002, 21, 1106–1112. 10.1021/IC00133A045.

[ref34] Maiti B. K.; Govil N.; Kundu T.; Moura J. J. G. Designed Metal-ATCUN Derivatives: Redox- and Non-Redox-Based Applications Relevant for Chemistry, Biology, and Medicine. iScience 2020, 23, 101792 10.1016/j.isci.2020.101792. 33294799 PMC7701195

[ref35] Lappin A. G.; Murray C. K.; Margerum D. W. Electron Paramagnetic Resonance Studies of Nickel(III)-Oligopeptide Complexes. Inorg. Chem. 1978, 17, 1630–1634. 10.1021/IC50184A048.

[ref36] Domergue J.; Guinard P.; Douillard M.; Pécaut J.; Proux O.; Lebrun C.; Le Goff A.; Maldivi P.; Delangle P.; Duboc C. A Bioinspired NiII Superoxide Dismutase Catalyst Designed on an ATCUN-like Binding Motif. Inorg. Chem. 2021, 60, 12772–12780. 10.1021/ACS.INORGCHEM.1C00899/SUPPL_FILE/IC1C00899_SI_001.PDF. 34416109

[ref37] Wezynfeld N. E.; Bonna A.; Bal W.; Frączyk T. Ni(Ii) Ions Cleave and Inactivate Human Alpha-1 Antitrypsin Hydrolytically, Implicating Nickel Exposure as a Contributing Factor in Pathologies Related to Antitrypsin Deficiency. Metallomics 2015, 7, 596–604. 10.1039/C4MT00316K. 25579632

[ref38] Bossu F. P.; Paniago E. B.; Margerum D. W.; Kirksey S. T.; Kurtz J. L. Trivalent Nickel Catalysis of the Autoxidation of Nickel(II) Tetraglycine. Inorg. Chem. 1978, 17, 1034–1042. 10.1021/IC50182A047.

[ref39] Levine J.; Etter J.; Apostol I. Nickel-Catalyzed N-Terminal Oxidative Deamination in Peptides Containing Histidine at Position 2 Coupled with Sulfite Oxidation. J. Biol. Chem. 1999, 274, 4848–4857. 10.1074/JBC.274.8.4848. 9988725

[ref40] Murray C. K.; Margerum D. W. Axial Coordination of Monodentate Ligands with Nickel(III) Peptide Complexes. Inorg. Chem. 1982, 21, 3501–3506. 10.1021/ic00139a046.

[ref41] Heering H. A.; Bulsink Y. B. M.; Hagen W. R.; Meyer T. E. Influence of Charge and Polarity on the Redox Potentials of High-Potential Iron-Sulfur Proteins: Evidence for the Existence of Two Groups. Biochemistry 1995, 34, 14675–14686. 10.1021/bi00045a008. 7578075

[ref42] Hosseinzadeh P.; Lu Y. Design and Fine-Tuning Redox Potentials of Metalloproteins Involved in Electron Transfer in Bioenergetics. Biochim. Biophys. Acta, Bioenerg. 2016, 1857, 557–581. 10.1016/j.bbabio.2015.08.006. PMC4761536 26301482

[ref43] Tonelli M.; Sacks F.; Pfeffer M.; Gao Z.; Curhan G. Relation between Serum Phosphate Level and Cardiovascular Event Rate in People with Coronary Disease. Circulation 2005, 112, 2627–2633. 10.1161/CIRCULATIONAHA.105.553198. 16246962

[ref44] Bergwitz C.; Jüppner H. Phosphate Sensing. Adv. Chronic Kidney Dis. 2011, 18, 132–144. 10.1053/j.ackd.2011.01.004. 21406298 PMC3059779

[ref45] Paleček E.; Bartošík M. Electrochemistry of Nucleic Acids. Chem. Rev. 2012, 112, 3427–3481. 10.1021/cr200303p. 22372839

[ref46] Sigel H.; Griesser R. Nucleoside 5′-triphosphates: self-association, acid-base, and metal ion-binding properties in solution. Chem. Soc. Rev. 2005, 34, 875–900. 10.1039/B505986K. 16172677

[ref47] Kaczmarek P.; Szczepanik W.; Jeżowska-Bojczuk M. Acid-Base, Coordination and Oxidative Properties of Systems Containing ATP, L-Histidine and Ni(II) Ions. Dalton Trans. 2005, 22, 3653–3657. 10.1039/b508962j. 16258616

[ref48] Sigel H. Interactions of Metal Ions with Nucleotides and Nucleic Acids and Their Constituents. Chem. Soc. Rev. 1993, 22, 255–267. 10.1039/CS9932200255.

[ref49] Alderighi L.; Gans P.; Ienco A.; Peters D.; Sabatini A.; Vacca A. Hyperquad Simulation and Speciation (HySS): A Utility Program for the Investigation of Equilibria Involving Soluble and Partially Soluble Species. Coord. Chem. Rev. 1999, 184, 311–318. 10.1016/S0010-8545(98)00260-4.

[ref50] Farkas E.; Sóvágó I.; Gergely A. Studies on Transition-Metal-Peptide Complexes. Part 8. Parent and Mixed-Ligand Complexes of Histidine-Containing Dipeptides. J. Chem. Soc., Dalton Trans. 1983, 8, 1545–1551. 10.1039/DT9830001545.

[ref51] Brookes G.; Pettit L. D. Thermodynamics of formation of complexes of copper(II) and nickel(II) ions with glycylhistidine, β-alanylhistidine, and histidylglycine. J. Chem. Soc., Dalton Trans. 1975, 74, 2112–2117. 10.1039/DT9750002112.

[ref52] Raics M.; Lihi N.; Laskai A.; Kállay C.; Várnagy K.; Sóvágó I. Nickel(Ii), Zinc(Ii) and Cadmium(Ii) Complexes of Hexapeptides Containing Separate Histidyl and Cysteinyl Binding Sites. New J. Chem. 2016, 40, 5420–5427. 10.1039/c6nj00081a.

[ref53] Turi I.; Kállay C.; Szikszai D.; Pappalardo G.; Di Natale G.; De Bona P.; Rizzarelli E.; Sóvágó I. Nickel(II) Complexes of the Multihistidine Peptide Fragments of Human Prion Protein. J. Inorg. Biochem. 2010, 104, 885–891. 10.1016/j.jinorgbio.2010.04.008. 20494446

[ref54] Wezynfeld N. E.; Goch W.; Bal W.; Frączyk T. Cis-Urocanic Acid as a Potential Nickel(Ii) Binding Molecule in the Human Skin. Dalton Trans. 2014, 43, 3196–3201. 10.1039/c3dt53194e. 24352502

[ref55] Solaini G. Spermine Antagonizes the Binding of Adriamycin to the Inner Membrane of Heart Mitochondria. Biochem. Biophys. Res. Commun. 1989, 159, 791–798. 10.1016/0006-291X(89)90064-8. 2930543

[ref56] Shullenberger D. F.; Eason P. D.; Long E. C. Design and Synthesis of a Versatile DNA-Cleaving Metallopeptide Structural Domain. J. Am. Chem. Soc. 1993, 115, 11038–11039. 10.1021/ja00076a091.

[ref57] Bal W.; Liang R.; Lukszo J.; Lee S.-H.; Dizdaroglu M.; Kasprzak K. S. Ni(II) Specifically Cleaves the C-Terminal Tail of the Major Variant of Histone H2A and Forms an Oxidative Damage-Mediating Complex with the Cleaved-Off Octapeptide. Chem. Res. Toxicol. 2000, 13, 616–624. 10.1021/tx000044l. 10898594

[ref58] Jin Y.; Lewis M. A.; Gokhale N. H.; Long E. C.; Cowan J. A. Influence of Stereochemistry and Redox Potentials on the Single- and Double-Strand DNA Cleavage Efficiency of Cu(II)· and Ni(II)·Lys-Gly-His-Derived ATCUN Metallopeptides. J. Am. Chem. Soc. 2007, 129, 8353–8361. 10.1021/ja0705083. 17552522

