# Supplementary material for: Tuning Receptor Properties of Metal–Amyloid Beta Complexes. Studies on the Interaction between Ni(II)–Aβ5–9 and Phosphates/Nucleotides
Source: Inorg Chem. 2021 Dec 8;60(24):19448–56. doi: 10.1021/acs.inorgchem.1c03285 (PMC8693174; doi:10.1021/acs.inorgchem.1c03285)
Supplement: Supplementary file 1 — ic1c03285_si_001.pdf [file ic1c03285_si_001.pdf]

Supplementary Information for

Tuning Receptor Properties of Metal-Amyloid Beta Complexes. Studies on the Interaction between Ni(II)-Aβ_{5-9} and Phosphates/Nucleotides

Aleksandra Tobolska,*[a],[b] Nina E. Wezynfeld,*[a] Urszula E. Wawrzyniak,[a] Wojciech Bal,[c] and Wojciech Wróblewski[a]

[a] Chair of Medical Biotechnology, Faculty of Chemistry, Warsaw University of Technology, Noakowskiego 3, 00-664 Warsaw (Poland)

[b] Faculty of Chemistry, University of Warsaw, Pasteura 1, 02-093 Warsaw (Poland)

[c] Institute of Biochemistry and Biophysics, Polish Academy of Sciences, Pawińskiego 5a, 02-106 Warsaw (Poland)

E-mail: atobolska@ch.pw.edu.pl, nwezynfeld@ch.pw.edu.pl

1. FIGURES AND TABLES

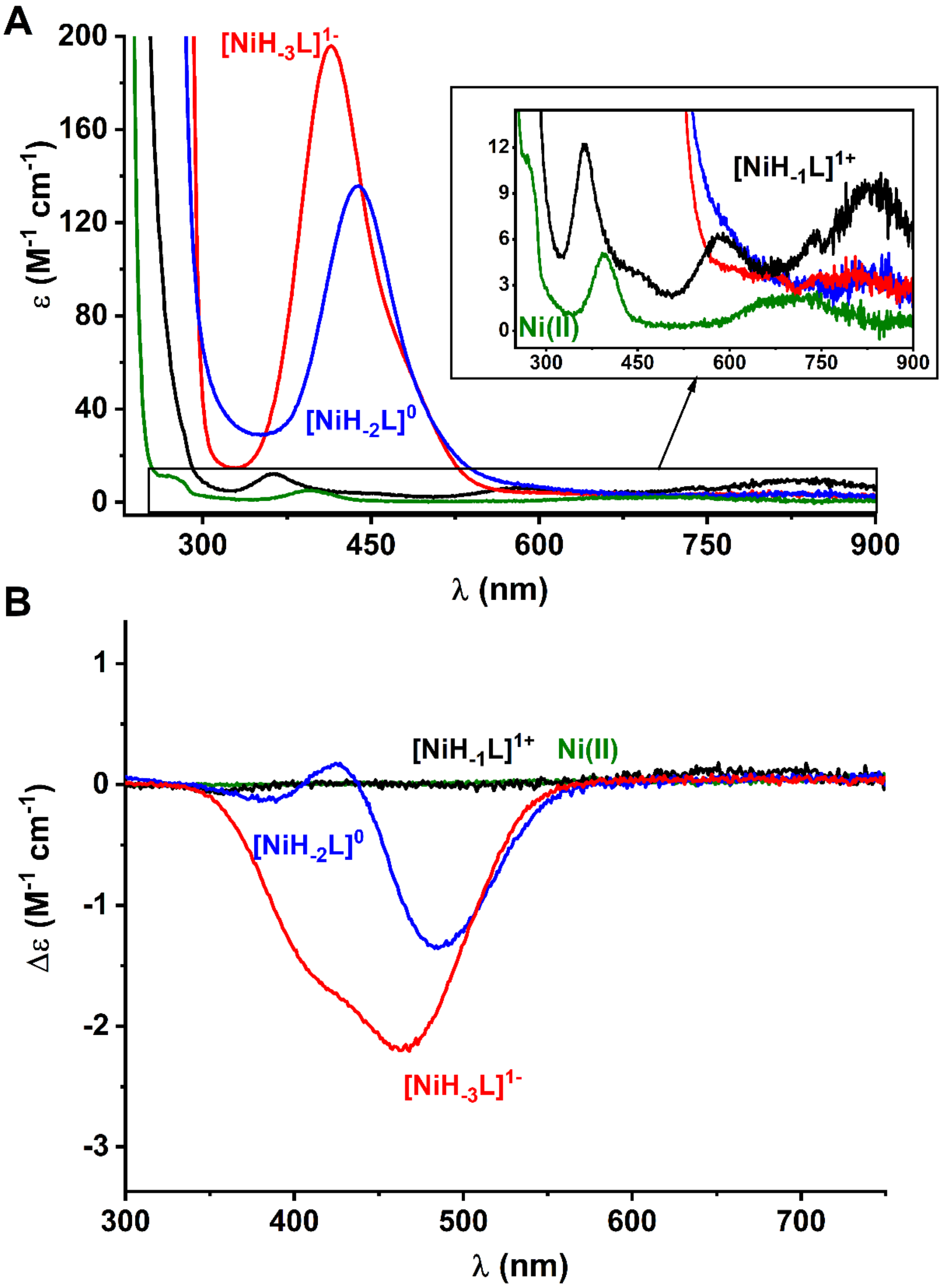

Figure S1. UV-vis (A) and CD (B) spectra of Ni(II)-Aβ$_{5-9}$ species calculated based on potentiometric data shown in Table 1 and spectroscopic titration data shown in Figure 2.

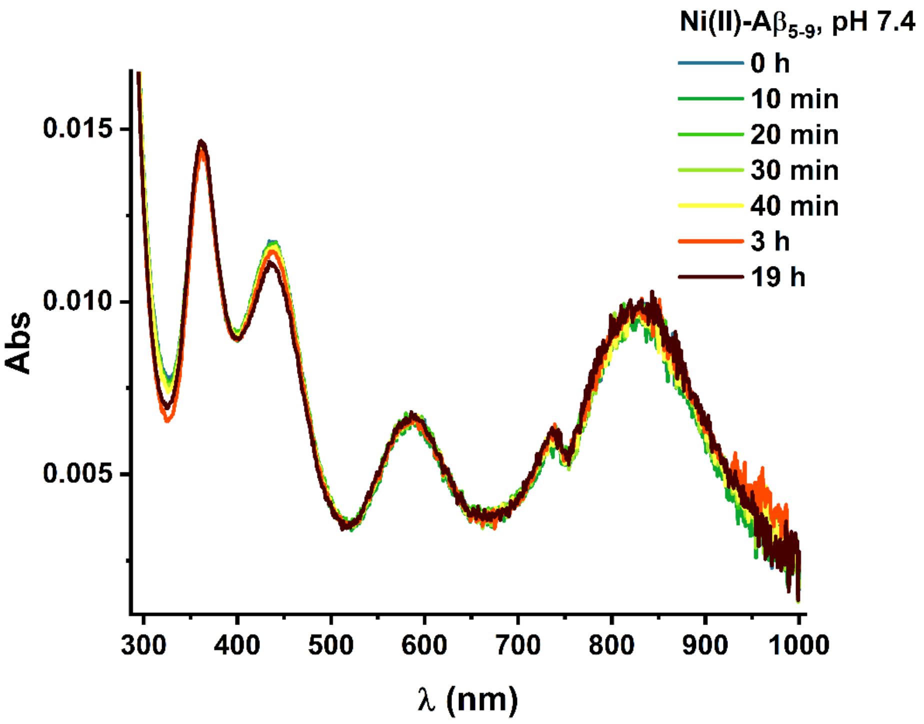

Figure S2. UV-vis spectra of 1.1 mM Aβ$_{5-9}$/1 mM Ni(II) at pH 7.4 monitored for nineteen hours. The "0 h" indicates the start of the UV-vis measurements after the reagent mixing and the pH adjustment that took about thirty minutes.

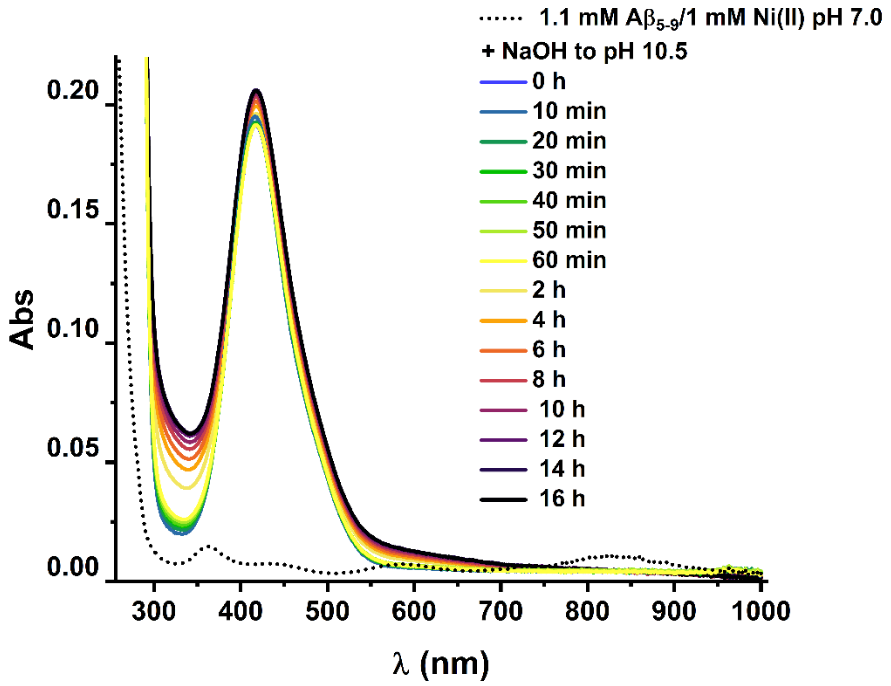

Figure S3. UV-vis spectra of 1.1 mM Aβ$_{5-9}$/1 mM Ni(II) after the increase of pH from 7.0 (a dotted black line) to pH 10.5 monitored for sixteen hours (solid lines). The "0 h" indicates the start of the UV-vis measurements after the pH adjustment to 10.5, which took about twenty minutes.

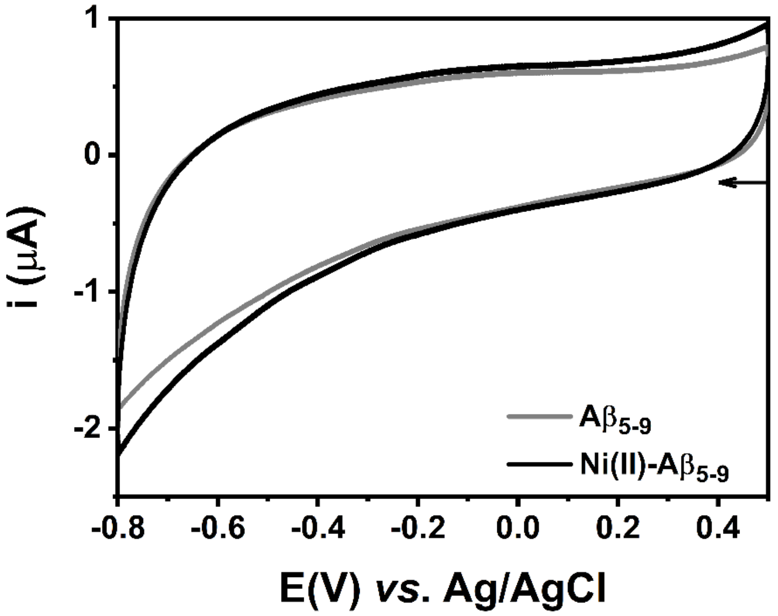

Figure S4. CV curves obtained for 0.5 mM Aβ$_{5-9}$ in the absence (a grey dashed line) and in the presence of 0.45 mM Ni(II) (a solid black line) recorded toward negative potentials in 100 mM KNO_3 at pH 7.4. Scan rate: v = 0.1 V/s.

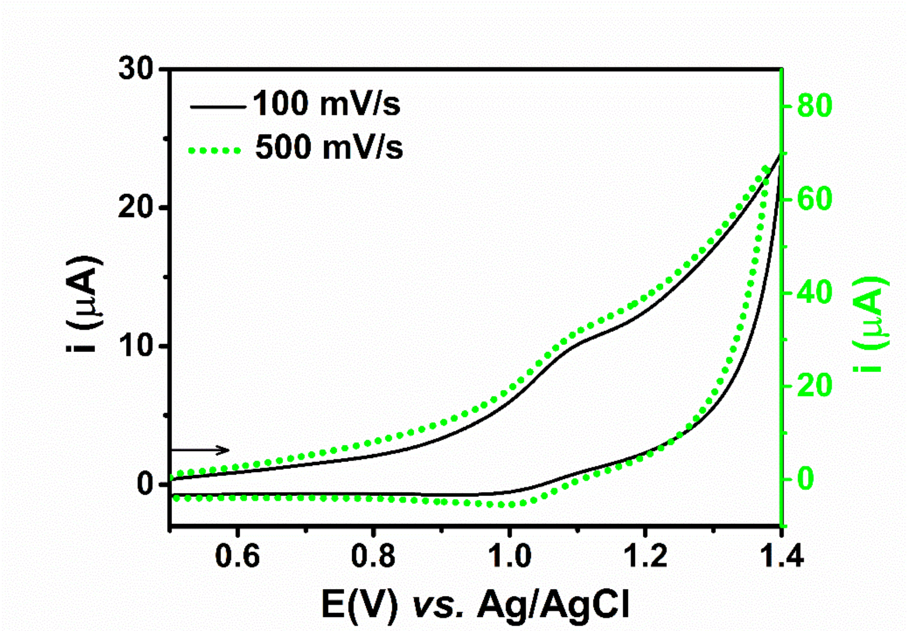

Figure S5. CV curves obtained for Ni(II)-Aβ$_{5-9}$ complex (0.9:1.0 molar ratio), recorded in 100 mM KNO_3 at pH 7.4, scan rates v = 0.1 V/s (a solid black curve) and v = 0.5 V/s (a dotted green curve).

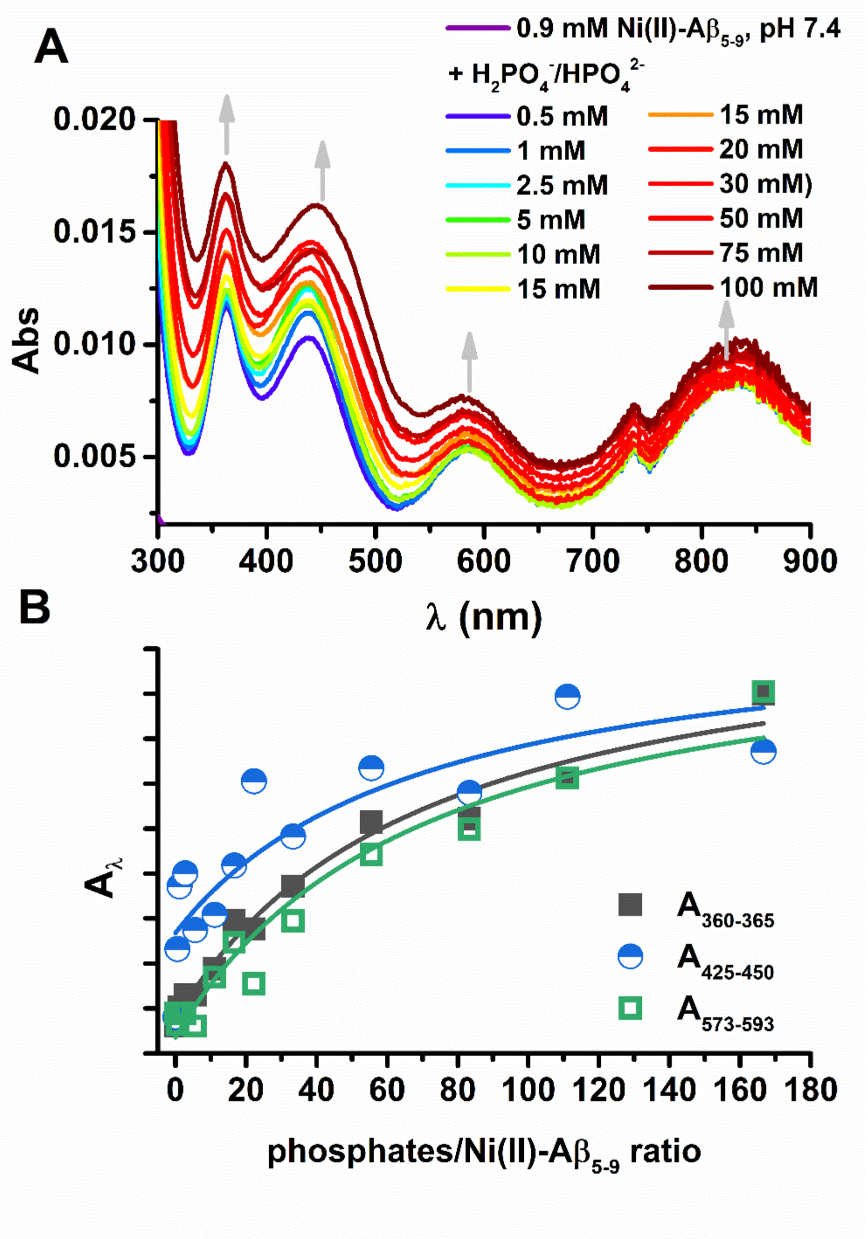

Figure S6. Spectra of the UV-vis titration of 0.9 mM Ni(II)-Aβ$_{5-9}$ with phosphates at pH 7.4 (A). The dependence of absorbance at selected wavelengths on phosphate/Ni(II)-Aβ$_{5-9}$ ratio derived from the spectra above (B).

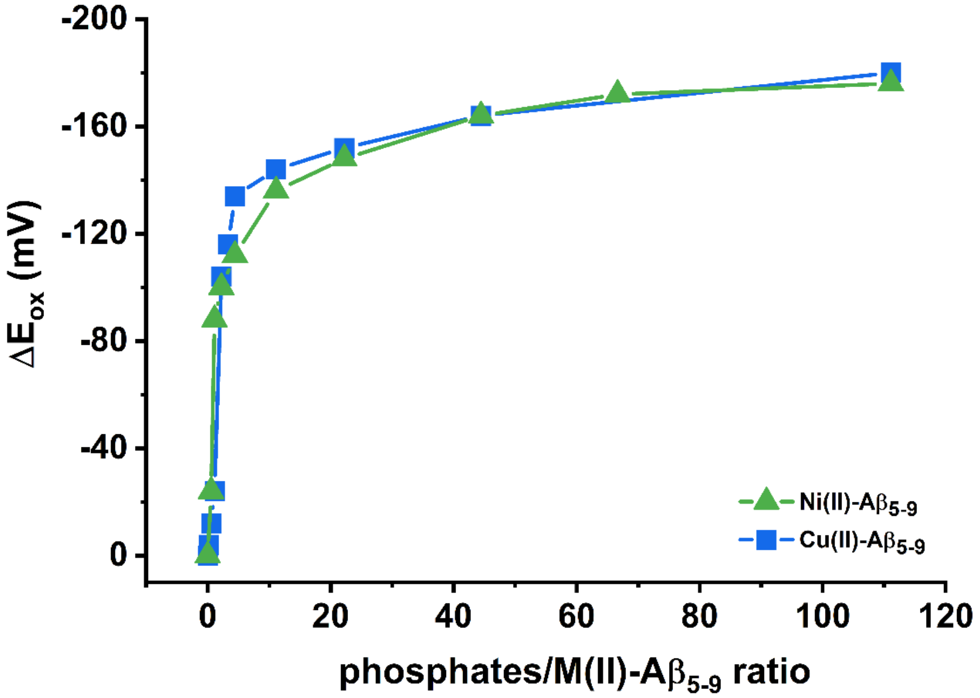

Figure S7. Dependence of ΔE_{ox} on the phosphates to metal ion ratio for $A\beta_{5-9}$ complexes with Cu(II) (a blue curve) and Ni(II) (a light green curve). ΔE_{ox} is the difference of the potential values of Cu(II) or Ni(II) oxidation of the ternary system with phosphate anions and the binary complex. The Cu(II) complexes were described in detail in our previous paper.[1] Concentrations of regents: 0.5 mM $A\beta_{5-9}$, 0.45 mM Cu(II) or Ni(II), 0-50 mM phosphate anions.

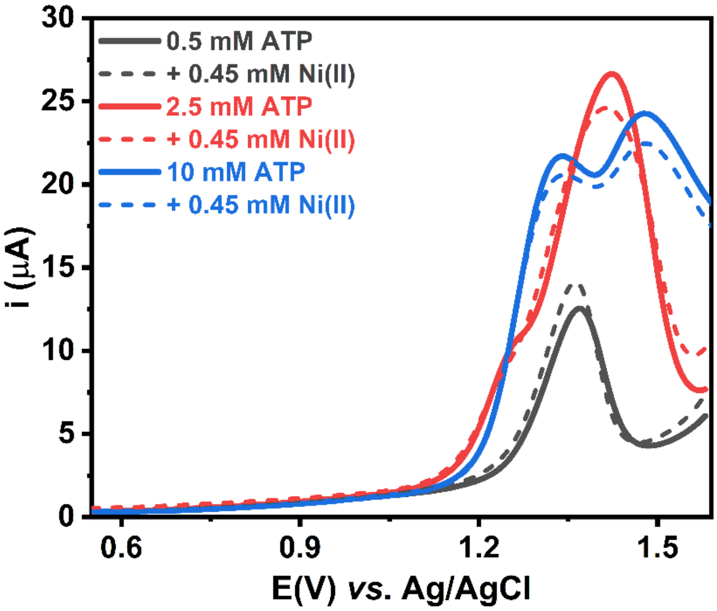

Figure S8. DPV curves obtained for different ATP concentrations in the absence (solid lines) and in the presence of 0.45 mM Ni(II) ions (dashed lines), recorded in 100 mM KNO_3 at pH 7.4.

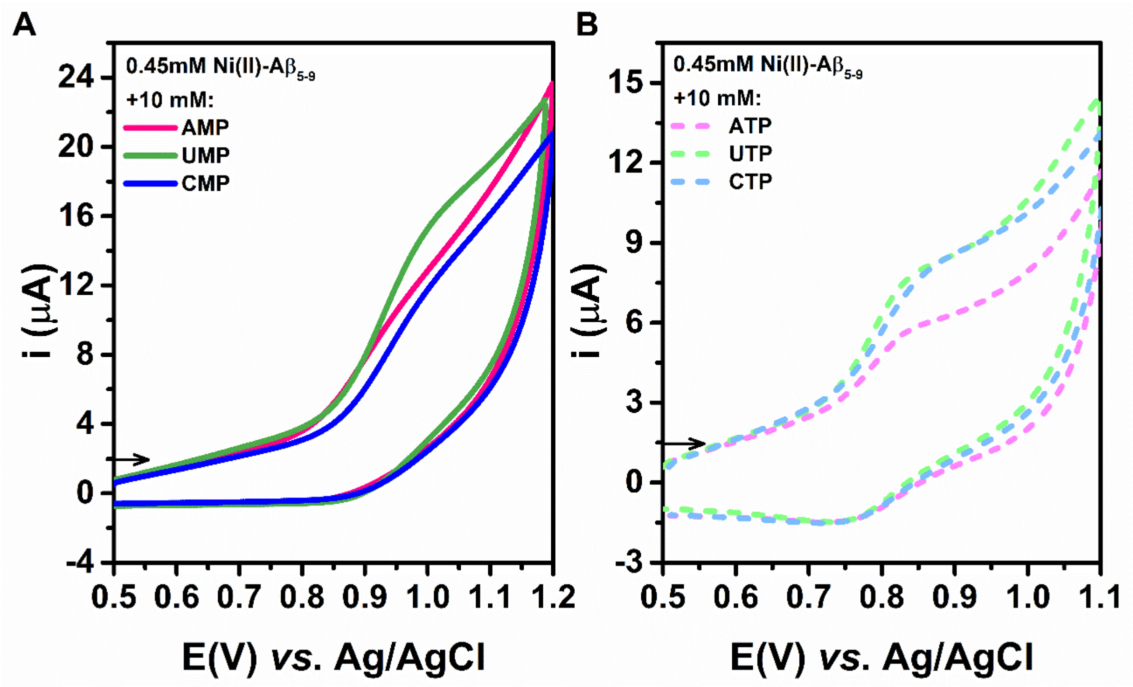

Figure S9. CV curves obtained for 0.45 mM Ni(II)-A$\beta_{5\text{-}9}$ complex after addition of 10 mM nucleoside monophosphates AMP, UMP, CTP – solid lines (A) or after addition of 10 mM nucleoside triphosphates ATP, UTP, CTP – dashed lines (B), recorded in 100 mM KNO_3 at pH 7.4. CV scan rate: v = 0.1 V/s.

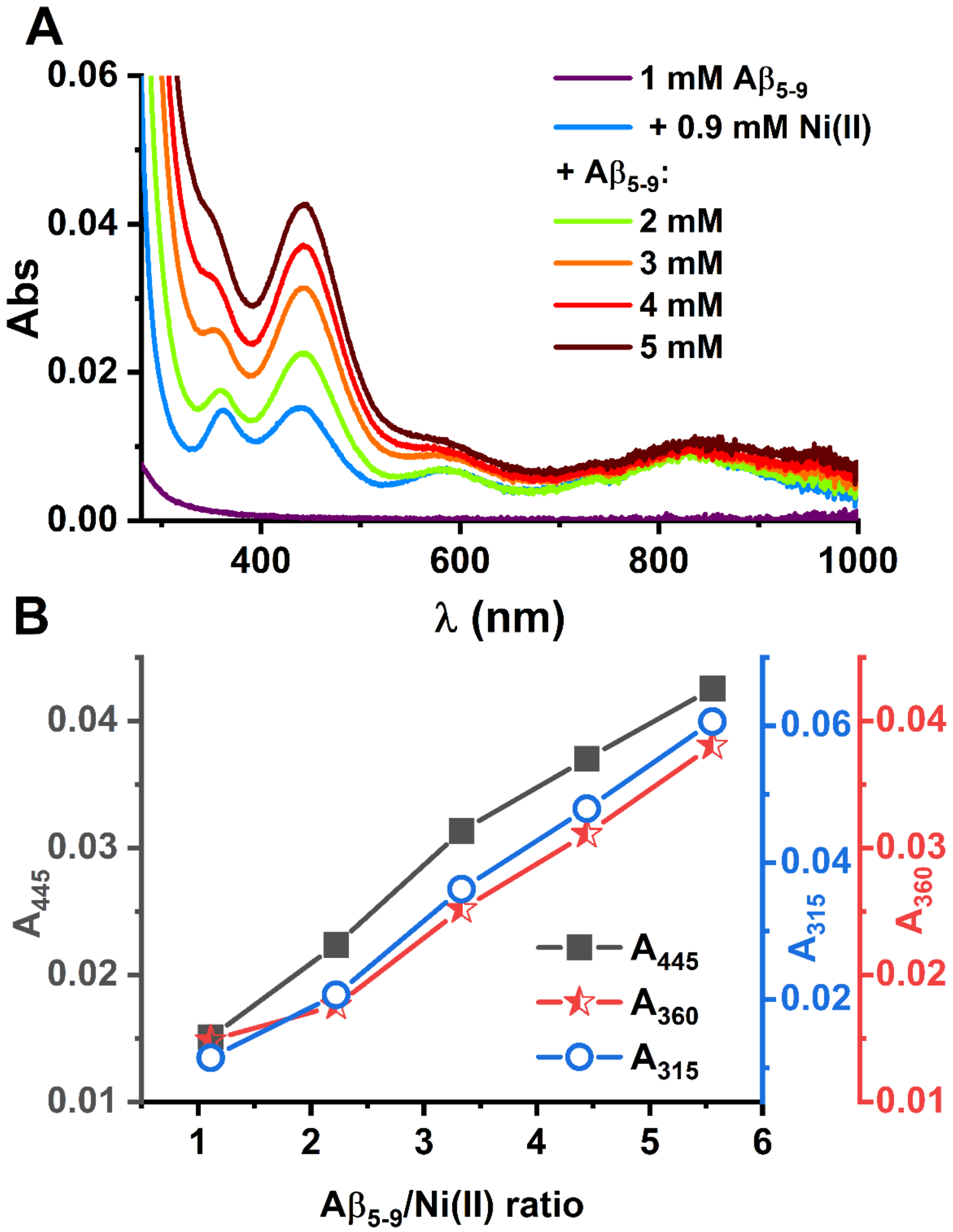

Figure S10. UV-vis spectra of the titration of 0.9 mM Ni(II)/1 mM A$\beta_{5\text{-}9}$ with A$\beta_{5\text{-}9}$ at pH 7.4 (A). The comparison of the changes in the absorbance of the studied Ni(II) complex bands at 360 nm and 420 nm with the absorbance at 315 nm representing the general changes in the UV region upon the peptide addition on A$\beta_{5\text{-}9}$/Ni(II) ratio (B).

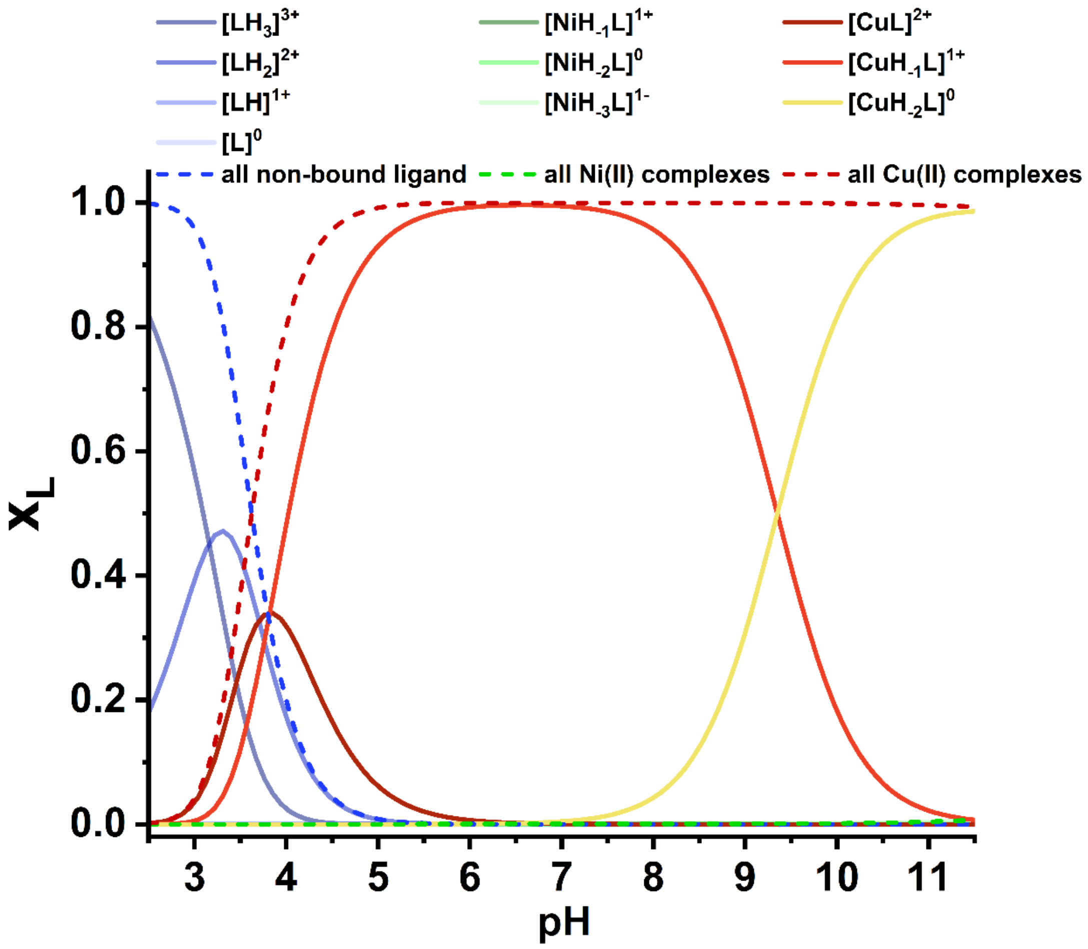

Figure S11. Ligand species distribution calculated for 1 mM of Cu(II), 1 mM Ni(II), 1 mM A$\beta_{5\text{-}9}$ based on the potentiometric constants given in Table 1 and in the literature. [2]

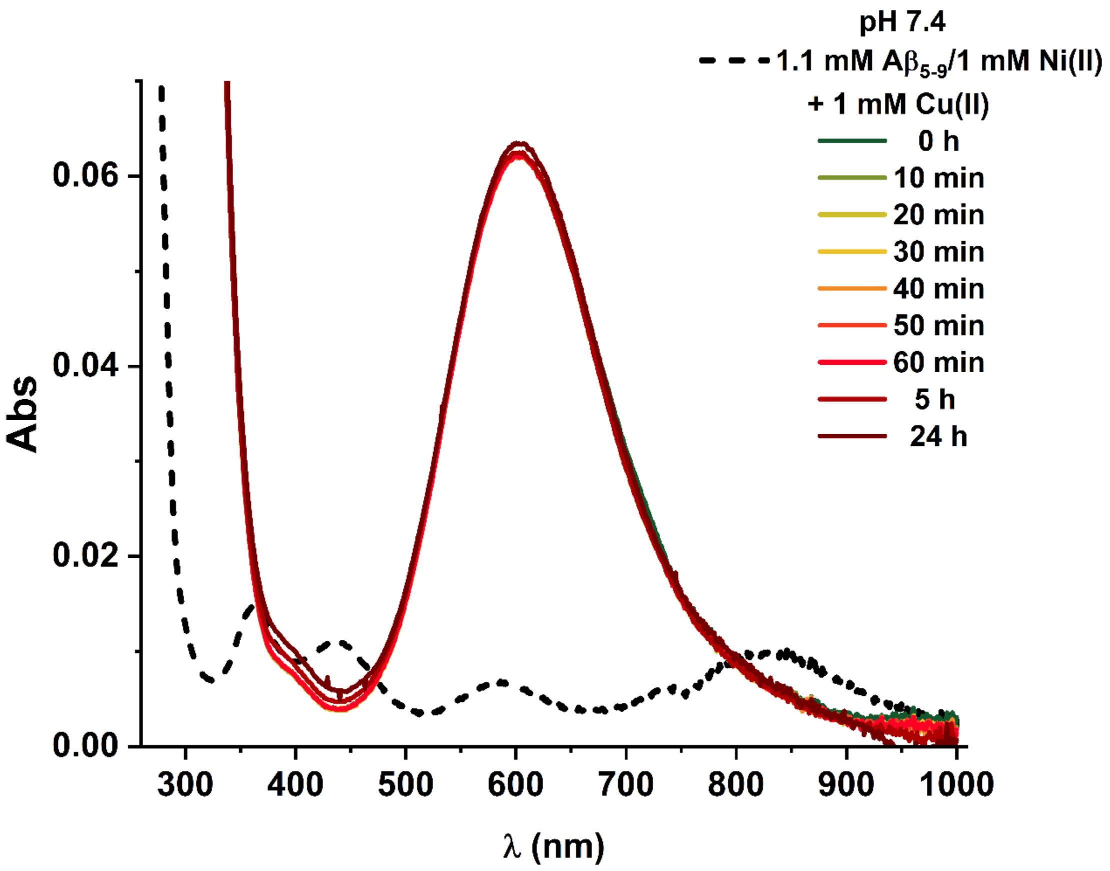

Figure S12. UV-vis spectra of 1.1 mM A$\beta_{5\text{-}9}$/1 mM Ni(II) at pH 7.4 alone and after addition of 1 mM Cu(II) monitored for twenty-four hours. The "0 h" indicates the start of the UV-vis measurements after the reagent mixing and the pH adjustment that took about fifteen minutes.

Table S1. Oxidation potential values determined from DPV curves of Ni(II)-Aβ_{5-9} in the presence of 10 mM anions and nucleotides at pH 7.4

**Ni(II)-Aβ_{5-9} in the presence of:**	**$E_{Ni(II)/Ni(III)}$ (V) *vs* Ag/AgCl ± SD**
Cl^-	1.008±0.002
SO_4^{2-}	0.995±0.008
CH_3COO^-	0.948±0.006
$H_2PO_4^-/HPO_4^{2-}$	0.860±0.002
AMP	0.890±0.006
UMP	0.912±0.008
CMP	0.912±0.008
ATP	0.786±0.004
UTP	0.788±0.002
CTP	0.824±0.016

2. REFERENCES

(1) Tobolska, A.; Wezynfeld, N. E.; Wawrzyniak, U. E.; Bal, W.; Wróblewski, W. Copper(II) Complex of N-Truncated Amyloid-β Peptide Bearing a His-2 Motif as a Potential Receptor for Phosphate Anions. *Dalt. Trans.* **2021**, *50* (8), 2726–2730.

(2) Wezynfeld, N. E.; Tobolska, A.; Mital, M.; Wawrzyniak, U. E.; Wiloch, M. Z.; Płonka, D.; Bossak-Ahmad, K.; Wroblewski, W.; Bal, W. Aβ5-x Peptides: N-Terminal Truncation Yields Tunable Cu(II) Complexes. *Inorg. Chem.* **2020**, *59*, 14000–14011.
